# LncRNAs‐circRNAs as Rising Epigenetic Binary Superstars in Regulating Lipid Metabolic Reprogramming of Cancers

**DOI:** 10.1002/advs.202303570

**Published:** 2023-11-08

**Authors:** Shanshan Liu, Benzheng Jiao, Hongguang Zhao, Xinyue Liang, Fengyan Jin, Xiaodong Liu, Ji‐Fan Hu

**Affiliations:** ^1^ Key Laboratory of Organ Regeneration and Transplantation of Ministry of Education Cancer Center, First Hospital Jilin University Changchun 130021 China; ^2^ Hematology Department First Hospital Jilin University Changchun 130021 China; ^3^ NHC Key Laboratory of Radiobiology (Jilin University) School of Public Health Jilin University Changchun 130021 China; ^4^ Nuclear Medicine Department First Hospital Jilin University Changchun 130021 China; ^5^ Radiation Medicine Department, School of Public Health and Management Wenzhou Medical University Wenzhou 325035 China; ^6^ Palo Alto Veterans Institute for Research Stanford University Medical School Palo Alto CA 94304 USA

**Keywords:** cancer, cholesterol, circRNAs, fatty acids, lipid metabolic reprogramming, lncRNAs

## Abstract

As one of novel hallmarks of cancer, lipid metabolic reprogramming has recently been becoming fascinating and widely studied. Lipid metabolic reprogramming in cancer is shown to support carcinogenesis, progression, distal metastasis, and chemotherapy resistance by generating ATP, biosynthesizing macromolecules, and maintaining appropriate redox status. Notably, increasing evidence confirms that lipid metabolic reprogramming is under the control of dysregulated non‐coding RNAs in cancer, especially lncRNAs and circRNAs. This review highlights the present research findings on the aberrantly expressed lncRNAs and circRNAs involved in the lipid metabolic reprogramming of cancer. Emphasis is placed on their regulatory targets in lipid metabolic reprogramming and associated mechanisms, including the clinical relevance in cancer through lipid metabolism modulation. Such insights will be pivotal in identifying new theranostic targets and treatment strategies for cancer patients afflicted with lipid metabolic reprogramming.

## Graphical abstract

The lipid metabolism‐related lncRNAs and circRNAs have emerged as rising epigenetic binary superstars in regulating lipid metabolic reprogramming of cancers. In this review, their regulatory targets, associated mechanisms, as well as clinical relevance are summarized to cast light on identifying new theranostic targets and treatment strategies for cancer patients afflicted with lipid metabolic reprogramming.

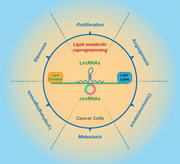

## 1 Introduction

Since the recognition of metabolic reprogramming as a crucial characteristic of cancer in 2011,^[^ ^1^ ^]^ numerous reviews have documented various features of metabolic reprogramming in different types of cancer.^[^ ^2^, ^3^, ^4^, ^5^, ^6^ ^]^ In response to the demands of uncontrolled proliferation and the stress derived from the surrounding microenvironment, such as hypoxia and nutrient deprivation, cancer cells require more glucose, glutamine, and fatty acids to support rapid ATP generation, increased biosynthesis of macromolecules, and redox homeostasis, compared to normal cells. Consequently, several metabolic phenotypes have emerged in cancer, including excessive glucose and/or glutamine uptake, increased aerobic glycolysis and lactate secretion, and alterations in lipid synthesis and degradation. These metabolic features are shaped by oncogenic stimuli, such as the inactivation of tumor suppressors or activation of oncogenes or imposed by the harsh tumor microenvironment.^[^ ^7^, ^8^, ^9^ ^]^

To cope with oncogenic and environmental stimuli, various metabolic enzymes and signaling pathways in regulating glucose, glutamine and lipid metabolism are activated or dysregulated. The hallmark of “aerobic glycolysis” or “Warburg effect” is the most important metabolic alteration in cancer, where cancer cells prefer glycolysis for energy and biosynthesis of biomass, rather than mitochondrial oxidative phosphorylation, even in the presence of oxygen. Subsequently, amino acid (e.g., glutamine, serine and glycine) and lipid (e.g., fatty acid and cholesterol) metabolism have also been identified as important metabolic aberrations for carcinogenesis, progression and metastasis. In addition to elevated expression and activation of metabolic enzymes and signaling pathways, accumulating evidences have been demonstrated that the non‐coding RNAs also respond to these oncogenic and environmental stimuli and play critical roles in metabolic reprogramming of cancer though diverse epigenetic regulatory mechanisms.^[^ ^8^, ^10^, ^11^ ^]^

With the rapid development of RNA‐seq technologies and bioinformatics analysis in the last decades, various non‐coding RNAs have been discovered to play pleiotropic functions in regulation of gene expression under physiological and pathologic conditions. Non‐coding RNAs are typically classified into three categories based on their sizes and structures: small non‐coding RNAs (less than 200 nucleotides), long non‐coding RNAs (lncRNAs, more than 200 nucleotides) and circular RNAs (circRNAs).^[^ ^11^, ^12^ ^]^ Regarding their molecular functions, microRNAs (miRNAs) are often utilized by argonaute proteins to either facilitate the degradation of targeted mRNAs or hinder their translation through full or partial complementarity. However, lncRNAs and circRNAs play diverse roles in regulating genes expression by interacting with DNA, RNA and proteins, including chromatin modification, transcriptional interference or activation as ceRNAs (competing endogenous RNAs) and scaffolds for protein complexes, even partially acing as protein/peptide translational templates.^[^ ^12^, ^13^, ^14^ ^]^ Increasing evidence has confirmed that non‐coding RNAs are often dysregulated but play as key epigenetic regulators in many hallmarks of cancer, including metabolic reprogramming, facilitating uncontrolled proliferation, invasion and metastasis of cancer cells by adjusting nutrient uptake and metabolic reactions. While non‐coding RNAs are well‐documented in glucose metabolism, there is less information on their roles in lipid metabolic reprogramming of cancer, especially lncRNAs and circRNAs.^[^ ^15^, ^16^ ^]^ Therefore, this review offers a comprehensive analysis of the regulatory functions exhibited by lncRNAs and circRNAs in the lipid metabolic reprogramming of cancer. Such insights will be pivotal in identifying new theranostic targets and treatment approaches for cancer patients afflicted with lipid metabolic reprogramming.

## 2 Overview of Lipid Metabolic Reprogramming in Cancer

Lipids in normal cells mainly consist of vast and complex groups of hydrophobic biomolecules, including fatty acids and cholesterols, which play a plethora of roles in bio‐membranes’ integrity and flexibility, energy storage and utilization, as well as protein modification and signal transduction.^[^ ^8^ ^]^ Lipid metabolism is a dynamic biological process that entails endogenous *de novo* synthesis and exogenous import of fatty acid and cholesterol, fatty acid β oxidation and cholesterol efflux, biogenesis and lipolysis of lipid droplets, and more.^[^ ^17^, ^18^ ^]^ However, the lipid metabolism is rewired in almost all types of cancer to support tumorigenesis, progression, metastasis, even stemness maintenance and chemotherapy resistance, which is mediated by various key lipid‐metabolic enzymes, transcriptional factors and signaling pathways in this dynamic process.^[^ ^5^, ^17^, ^19^, ^20^ ^]^

### 2.1 Endogenous de Novo Synthesis and Exogenous Import of Fatty Acid and Cholesterol in Cancer

**Figure 1:**
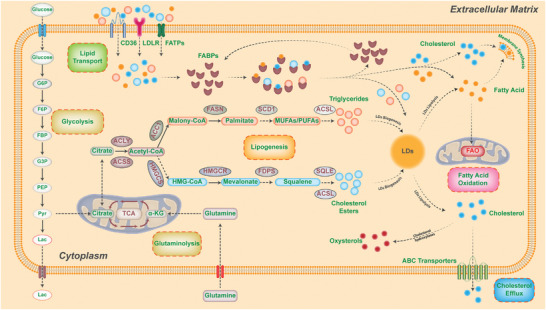
Rewiring of lipid metabolism in cancer. Lipid metabolism is a dynamic biological process that involves the endogenous *de novo* synthesis, exogenous import of fatty acids and cholesterol, fatty acid β oxidation, cholesterol efflux, biogenesis, and lipolysis of lipid droplets. Intracellular *de novo* lipogenesis begins with acetyl‐coenzyme A (acetyl‐CoA) derived from acetate by ATP‐citrate lyase (ACLY) or citrate by acetyl‐CoA synthetase (ACSS). Fatty acid synthesis requires acetyl‐CoA carboxylation into malonyl‐CoA by acetyl‐CoA carboxylases (ACC1/2), followed by the condensation of seven malonyl‐CoA molecules and one acetyl‐CoA molecule into the saturated 16‐carbon palmitate (16:0) by fatty acid synthase (FASN). Palmitate is then desaturated by stearoyl‐CoA desaturases (SCD) or elongated by fatty acid elongases (ELOVL) to form the monounsaturated 16‐carbon palmitoleate (16:1 n‐7) or 18‐carbon oleate (18:1 n‐9). Biogenesis of cholesterol also begins with acetyl‐CoA via the mevalonate pathway, which results in the synthesis of squalene and finally, cholesterol. Cancer cells can acquire fatty acids and cholesterol from various extracellular sources, such as LDL particles or fatty acid transport proteins. When lipids accumulate, cancer cells use these lipids to meet their energy consumption demand and redox homeostasis through fatty acid oxidation or β‐oxidation. Excess cholesterol is exported to the blood or converted into oxysterols through oxidation processes. Surplus fatty acids are esterified with glycerol or cholesterol into triglycerides and cholesteryl esters, which are incorporated into lipid droplets (LDs). When energy or membrane synthesis is needed, lipid droplets can be rapidly lipolyzed into free fatty acids and cholesterols to facilitate cancer cell proliferation and progression.

**Figure 2:**
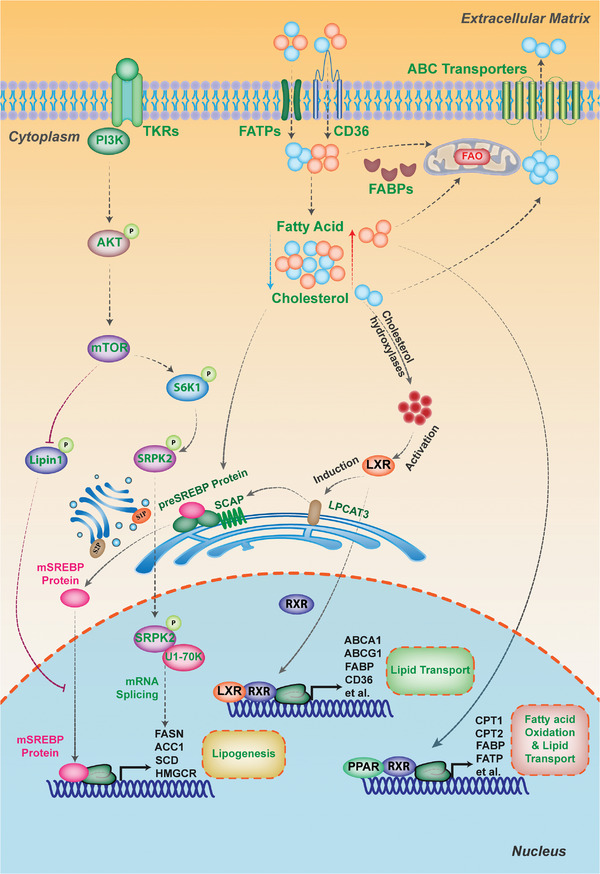
Transcriptional factors and oncogenic signaling pathways in lipid metabolism of cancer. Sterol regulatory element‐binding proteins (SREBPs) act as transcriptional factors that control the expression of most lipogenic enzymes involved in cholesterol and fatty acid biosynthesis. When lipid levels decrease, SREBPs are released from the SCAP‐INSIG complex in the endoplasmic reticulum and translocate to the Golgi, where they are cleaved by site‐1 and site‐2 proteases to release their active N terminus (mature SREBPs). Mature SREBPs move into the nucleus and bind to sterol response elements (SRE) in downstream target gene promoters to initiate transcription. The PI3K‐AKT‐mTOR pathway is frequently dysregulated in human cancers and can be activated by growth factor receptor tyrosine kinases (RTKs). The mTOR complexes participate in lipogenesis regulation through SREBP‐dependent or independent mechanisms. The mTOR‐dependent sequestration of Lipin‐1 in the cytoplasm enhances SREBP‐transcriptional activity in the nucleus, while the mTORC1/S6K1/SRPK2/U1‐70K axis increases mRNA splicing of lipogenic genes, such as *FASN* and *ACLY*. Liver X receptor (LXR) is an additional regulator of lipogenesis and a nuclear transcription factor receptor that senses oxysterols, cholesterol derivatives, to form the LXR‐RXRα complex. This complex induces the expression of genes involved in cholesterol efflux, such as *ABCA1*, and several lipogenic genes, including *FASN* and *SCD*. Peroxisome proliferator‐activated receptors (PPARs) are regulators of lipid metabolism and play vital roles in lipid β‐oxidation and storage in harsh environments when cellular energy is needed.

While most lipids in normal somatic cells come from fatty acids and cholesterols that derive from either dietary uptake or synthesis from hepatocytes and adipocytes,^[^ ^21^, ^22^ ^]^ various cancers prefer to thrive on intracellular *de novo* lipogenesis rather than utilizing exogenous sources under lipids‐constrained conditions. However, upon hypoxia or in adipose‐rich environments, these cancers switch toward extracellular lipids uptake (**Figures** **1** and **2**).^[^ ^23^, ^24^, ^25^, ^26^ ^]^

Intracellular *de novo* lipogenesis begins with acetyl‐coenzyme A (acetyl‐CoA), derived from acetate by ATP‐citrate lyase (ACLY) or citrate by acetyl‐CoA synthetase (ACSS), thereby connecting to other metabolic pathways like glucose and glutamine metabolism (Figure 1).^[^ ^27^, ^28^ ^]^ For *de novo* synthesis of fatty acids, acetyl‐CoA is initially converted into malonyl‐CoA by acetyl‐CoA carboxylases (ACC1/2), which is an essential, irreversible, and rate‐limiting step of *de novo* fatty acid. ACC1/2 has been identified to be active and highly expressed in several human cancers.^[^ ^29^, ^30^ ^]^ Subsequently, fatty acid synthase (FASN) condenses one molecule of acetyl‐CoA and seven malonyl‐CoA molecules into the saturated 16‐carbon palmitate (16:0). FASN is also an important but dysregulated lipid‐metabolic enzyme in many human epithelial cancers and strongly relevant with malignant progression and poor prognosis of tumors.^[^ ^31^, ^32^, ^33^ ^]^ Palmitate is then desaturated by stearoyl‐CoA desaturases (SCD) or/and elongated by fatty acid elongases (ELOVL) to form the monounsaturated 16‐carbon palmitoleate (16:1 n‐7) or 18‐carbon FA oleate (18:1 n‐9), which will provide key cornerstones for the generation of complex lipids like phospholipids and glycolipids.^[^ ^34^, ^35^, ^36^ ^]^ Thereinto, SCD1 overexpression has been demonstrated to contribute tumor development, inactivate the responses to sorafenib treatment, and associate with poor disease‐free survival in patients with hepatocellular carcinoma (HCC).^[^ ^19^, ^37^ ^]^

Concerning the *de novo* biogenesis of cholesterols, it also originates from acetyl‐CoA but goes through the mevalonate pathway (Figure 1). In this pathway, HMG‐CoA synthase (HMGCS) catalyzes the formation of 3‐hydroxy‐3‐methylglutaryl (HMG)‐CoA by condensing acetyl‐CoA and acetoacetyl‐CoA, which subsequently is reduced into mevalonate by HMG‐CoA reductase (HMGCR). The mevalonate subsequently undergoes a series of reactions to form the isoprenoid farnesyl pyrophosphate (FPP), which is then synthesized into squalene, and finally converted to cholesterol. Thereinto, the HMGCR and squalene epoxidase (SQLE) are the rate‐limiting enzymes of this process, and have been found to be dysregulated in multiple types of cancer, including prostate and breast cancers, gastric and colorectal cancers, and are positively associated with the growth and migration, radioresistance and poor prognosis of these cancers.^[^ ^38^, ^39^, ^40^, ^41^ ^]^ Alternatively, FPP can be used to produce geranylgeranyl‐pyrophosphate (GGPP), both of which are substrates for the prenylated modification of proteins, such as Rho GTPases prenylation, and synthesis of dolichol and ubiquinone (coenzyme Q10).^[^ ^42^, ^43^, ^44^ ^]^

Apart from *de novo* biosynthesis, fatty acids and cholesterols can be obtained from several extracellular sources (Figure 1). For instance, cholesterol from external sources is chiefly acquired through a process of endocytosis mediated by low‐density lipoprotein receptor (LDLR) or scavenger receptor B1 (SR‐B1), involving plasma LDL particles. Free fatty acids can also be absorbed into cancer cells via overexpression of fatty acid translocase (CD36) or fatty acid transport proteins (FATPs). Additionally, the upregulation of fatty acid‐binding proteins (FABPs) in cancer cells has been found to aid in the uptake of exogenous fatty acids,^[^ ^45^, ^46^, ^47^, ^48^ ^]^ These receptors and transporters have been identified as critical factors in the proliferation, metastasis, and epithelial‐mesenchymal transition (EMT) of various types of cancer, including glioblastoma, breast cancer, and HCC.^[^ ^49^, ^50^, ^51^, ^52^ ^]^

### 2.2 Fatty Acid Oxidation and Cholesterol Efflux in Cancer

As the fatty acids and cholesterols from either *de novo* biogenesis or exogenous uptake are increasingly accumulated, cancer cells can utilize these lipids to meet their demands of energy consumption and redox homeostasis via fatty acid oxidation (FAO) or β‐oxidation in mitochondria and peroxisome under harsh tumor microenvironment (Figure 1).^[^ ^5^, ^18^ ^]^ FAO is a repeated catabolic process of fatty acids shortening, with each cycle shortening two carbons from fatty acids to produce ATP, NADH and FADH2. The NADH and FADH2 can fuel the electron transport chain (ETC) of mitochondria to produce ATP, except for maintaining redox power.^[^ ^53^ ^]^ During this process, the carnitine palmitoyltransferase (CPT) system plays a critical role in transportation of fatty acids, composed of CPTI, CPTII, carnitine acylcarnitine translocase (CACT), and carnitine acetyltransferase (CRAT). All of them have been shown to be dysregulated in various cancers, promoting the resistance of cancer cells to energy stress.^[^ ^54^, ^55^ ^]^ For instance, as one of the rate‐limiting enzymes in FAO, CPTI can convert FA‐CoA into the carnitine derivatives to enter into the routes of mitochondrial and peroxisomal β‐oxidation.^[^ ^56^, ^57^ ^]^ No surprisingly, it has been found to be highly correlated with poor prognosis of patients with acute myeloid leukemia (AML) or ovarian cancer.^[^ ^36^, ^53^ ^]^

In addition, surplus cholesterol can be exported to the bloodstream via ATP‐binding cassette (ABC) transporters or converted into oxysterols via oxidation processes (Figure 1). These oxysterols then directly activate the transcription factors Liver X Receptor (LXR), promoting ABCA1 and ABCG1 expressions and E3 ubiquitin ligase‐mediated LDLR degradation, thereby reducing the intracellular excessive cholesterols.^[^ ^58^, ^59^, ^60^ ^]^ Recently, ABC transporters have been found to be overexpressed in multiple cancers, contributing dissemination and metastasis of cancer cells, and associated with resistance to a plethora of drugs.^[^ ^61^ ^]^ Additionally, LXRs and their ligands have also been found to be upregulated in various cancers, such as prostate carcinoma, breast and ovaries carcinoma, and multiple myeloma, playing vital roles in the proliferation and survival of cancer cells.^[^ ^62^ ^]^

### 2.3 Biogenesis and Lipolysis of Lipid Droplets in Cancer

Besides FAO and cholesterol efflux, excess fatty acids are esterified with glycerol or cholesterol into triglycerides (TGs) and cholesteryl esters (CEs), furtherly incorporated into lipid droplets (LDs), which are cytoplasmic organelles for energy storage, redox homeostasis and entrapment of anticancer drugs in cancer cells (Figure 1).^[^ ^63^, ^64^, ^65^ ^]^ The synthesis of TGs and CEs is usually conducted by endoplasmic reticulum (ER)‐resident enzymes: diacylglycerol acyltransferases (DGAT1 and DGAT2) that are involved into the synthesis of TGs, CEs are esterified by acyl‐coenzyme A:cholesterol O‐acyltransferases (ACAT1 and ACAT2).^[^ ^17^, ^66^ ^]^ These enzymes have also been found to be associated with malignant phenotypes of several cancers. For instance, ACAT1 has been identified to be overexpressed in glioblastomas, prostate or pancreas cancers, exerting pro‐tumorigenesis function, and is positively correlated with poor survival of these patients.^[^ ^67^, ^68^ ^]^

When energy or membrane synthesis is required, LDs can be rapidly lipolyzed into free fatty acids and cholesterols to facilitate proliferation and progression of cancer cells. This process is primarily catalyzed by various lipases and activators, including adipose triglyceride lipase (ATGL), hormone‐sensitive lipase (HSL), and monoglyceride lipase (MGLL) (Figure 1). Thereinto, ATGL is a rate‐limiting enzyme but its roles are complex in different types of cancer.^[^ ^69^, ^70^ ^]^ For instance, ATGL can promote proliferation and invasiveness in prostate, lung and colorectal cancer cells, but acts as a suppressor of malignancy in other types of cancers.^[^ ^70^, ^71^ ^]^ More interestingly, breast cancer cells can obtain free fatty acids to promote autologous proliferation and migration via ATGL‐dependent lipolysis in both itself and adipocytes in the local environment.^[^ ^72^ ^]^

### 2.4 Transcriptional Factors and Oncogenic Signaling Pathways in Lipid Metabolism of Cancer

As transcriptional factors, the sterol regulatory element‐binding proteins (SREBPs) play a key role in controlling most lipogenic enzymes’ expression at the transcriptional level, involving in cholesterol and fatty acid biosynthesis (Figure 2). Under normal conditions with sufficient intracellular lipid levels, SREBP proteins are translated as precursors and retained in the ER membrane by binding to the SREBP cleavage‐activating protein (SCAP)‐insulin induced gene (INSIG) complex. However, when the lipid levels decrease, SREBPs are released and translocated from SCAP‐INSIG complex in ER to the Golgi. Subsequently, the site‐1 and site‐2 proteases cleave SREBPs, producing their active N terminus (mature SPRBPs). These mature SPRBPs then enter the nucleus to bind to sterol response elements (SRE) in the promoters of downstream target genes and initiate the transcription. SREBP proteins come into three isoforms, with SREBP‐1a and SPREBP‐1c primarily governing fatty acids and TG's biosynthesis, while SREBP‐2 selectively facilitates the expression of enzymes involved in cholesterol biosynthesis.^[^ ^73^, ^74^ ^]^ These processes are highly regulated by intracellular levels of sterols, status of PI3K/Akt /mTOR signaling, and extracellular insulin and growth factors.^[^ ^5^ ^]^ which are always dysregulated in various cancers, including glioblastomas, breast cancer and HCC.^[^ ^75^, ^76^, ^77^, ^78^, ^79^ ^]^

Liver X receptor (LXR), another regulator of lipogenesis, is a nuclear transcription factor receptor that regulates many genes involved in fatty acid and cholesterol homeostasis (Figure 2). LXR acts as a sensor of oxysterols, derivatives of cholesterol, to form the LXR‐RXRα (retinoid X receptor α) complex. This complex then induces the expression of genes involved in cholesterol efflux such as ABCA1 and several lipogenic genes such as FASN and SCD.^[^ ^80^ ^]^ Therefore, using antagonists against LXR may be a new anti‐cancer choice. For instance, the tumor growth of glioblastoma, breast cancer or prostate cancer was significantly inhibited by synthetic LXR agonists GW3965 and T0901317 in vivo.^[^ ^52^, ^81^ ^]^ In addition, peroxisome proliferator‐activated receptors (PPARs), another regulator of lipid metabolism, play key roles in lipid β‐oxidation and storage. However, their roles in cancer metabolism are not fully understood.^[^ ^80^ ^]^

In addition to these nuclear transcription factors, some oncogenic signaling pathways take part in regulating lipid metabolism to shape the tumors’ unique lipidome.^[^ ^45^ ^]^ The PI3K‐AKT‐mTOR pathway, which is regularly disrupted in a variety of human cancers, can be induced by activating growth‐factor receptor tyrosine kinases (RTKs), as shown in Figure 2.^[^ ^82^ ^]^ When this pathway was activated, AKT is primarily involved in two crucial processes that fuel *de novo* lipid synthesis: transportation of metabolic intermediates to provide carbon sources and the production of reducing equivalents in the form of NADPH.^[^ ^83^ ^]^ Additionally, mTOR (mammalian target of rapamycin) complexes play a vital role in regulating lipogenesis with both SREBP‐dependent and SREBP‐independent pathways: sequestrating Lipin‐1 in the cytoplasm, which enhances SREBP‐transcriptional activity in the nucleus, and increasing mRNA splicing of lipogenic genes like FASN, ACLY, and ACSS2 via the mTORC1‐S6 kinase 1 (S6K1)‐serine/arginine protein kinase 2 (SRPK2)‐U1 small nuclear ribonucleoprotein 70 kDa (U1‐70K) axis.^[^ ^77^, ^84^ ^]^

## 3 Sketching the Potential Regulatory Mechanism of lncRNAs‐circRNAs in Gene Expression

**Figure 3:**
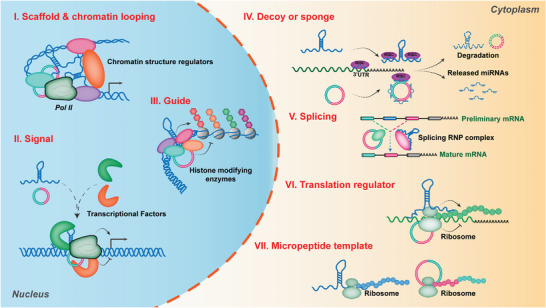
Potential mechanisms of lncRNAs & circRNAs in regulating gene expression. I) Regulation of genes transcription as a scaffold via binding with chromatin structure regulators; II) Regulation of gene transcription as a signal via recruiting transcriptional factors on promoter of target genes; III) Regulation of gene transcription as a guide via binding with histone modifying enzymes; IV) Decoying miRNAs as sponges; V) Modulating splicing of preliminary target mRNAs; VI) regulating target mRNA translation via binding with ribosome; VII) Functioning as templates to translate into micropeptides.

Based on their subcellular localizations, lncRNAs and circRNAs play distinct regulatory roles in the expression of target genes^[^ ^8^, ^9^, ^10^, ^85^ ^]^ (**Figure** **3**). In the nucleus, lncRNAs and circRNAs can act as scaffolds, signals or guides to modulate chromatin conformation, transcription factor recruitment or histone modification status.^[^ ^86^ ^]^ For instance, lncRNA *HOTTIP* can bind the 5′ regions of several *HOXA* genes cluster to form chromatin looping, which recruits the WDR5‐MLL histone methyltransferase complex to the promoters of these genes, thereby facilitating gene expression through H3K4me3 in mouse haematopoietic stem cells.^[^ ^87^ ^]^ As a pluripotency‐associated lncRNA, *lncPRESS1* can bind Sirtuin 6 to facilitate the transcription of pluripotency‐related genes with transcription‐permissive H3 acetylated at Lys56 (H3K56ac) and H3K9ac in human ESCs.^[^ ^88^ ^]^ EIciRNAs (exon‐intron circRNAs), such as *circEIF3J* and *circPAIP2*, can interact with U1 snRNA and Pol II to form transcription complex at the promoter, thereby enhancing parental gene transcription.^[^ ^89^ ^]^

In the cytoplasm, lncRNAs and circRNAs modulate the stability, splicing and translation of target gene mRNAd via RNA‐protein or RNA‐RNA interactions, such as decoying miRNAs as sponges, modulating splicing of preliminary target mRNAs, and regulating of mRNA translation via binding with ribosome. A number of lncRNAs and circRNAs bearing microRNA (miRNA)‐complementary sites can act as competitive endogenous RNAs or “sponges” of miRNAs to enhance the expression of target mRNAs, such as the lncRNA‐*PNUTS*/ miR‐205/ ZEB1and ZEB2 axis in the migration and invasion of breast cancer cell,^[^ ^90^ ^]^ and the *circEZH2*/ miR‐133b/IGF2BP2 axis in colorectal cancer progression.^[^ ^91^ ^]^ Moreover, lncRNAs and circRNAs can directly or indirectly facilitate the alternative splicing of target genes,^[^ ^92^ ^]^ such as *ZEB2‐anti* (ZEB2‐antisense RNA) in the regulation of *ZEB2* pre‐mRNA alternative splicing and translation,^[^ ^93^ ^]^ and the *circRAPGEF5/*RBFOX2 splicing axis in the formation of TFRC with exon‐4 skipping.^[^ ^94^ ^]^ Furthermore, *HOXB‐AS3* has been found to regulate ribosomal RNA transcription and *de novo* protein synthesis via binding and guiding EBP1 to the ribosomal DNA locus.^[^ ^95^ ^]^ In addition, a novel 161‐amino‐acid protein encoded by *circRsrc1* has been recently identified to bind mitochondrial protein C1qbp to modulate the assembly of mitochondrial ribosomes during spermatogenesis.^[^ ^96^ ^]^

Interestingly, some lncRNAs and circRNAs have the potentials to be translated into micropeptides in cap‐dependent, or IRES‐dependent, or m6A‐dependent manners. For instance, *circ‐AKT3* holds the potential to encode a novel 174 amino acid (aa) protein, which inhibits the proliferation, radiation resistance and in vivo tumorigenicity of GBM cells via interacting with phosphorylated PDK1, thereby modulating the PI3K/AKT signal intensity.^[^ ^97^ ^]^ Moreover, consensus m6A motifs has been found to be enriched in circRNAs, which can drive translation initiation with initiation factor eIF4G2 and m6A reader YTHDF3.^[^ ^98^ ^]^ Certain lncRNAs and circRNAs distribute in both cellular chambers, and they can shuttle between cytoplasm and nucleus to play diverse roles in regulating the expression of target genes. For example, with the help of two RBPs, HuR and GRSF1, nuclear DNA‐encoded lncRNA *RMRP* was imported into mitochondria to maintain structure and mediate oxidative phosphorylation and mitochondrial DNA replication.^[^ ^99^ ^]^ *LINC00473* has been identified to shuttle between mitochondria and lipid drops to modulate lipolysis and mitochondrial oxidative functions in human thermogenic adipocytes.^[^ ^100^ ^]^

## 4 LncRNAs‐circRNAs Regulate the Rewiring of Lipid Metabolism in Cancer

**Figure 4:**
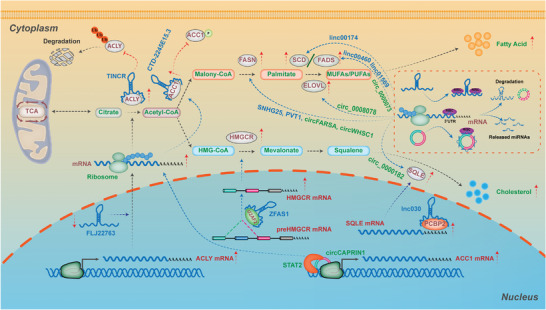
The lncRNAs & circRNAs in regulating lipogenesis of cancer. During the process of *de novo* lipogenesis, there are multiple rate‐limiting enzymes modulated by various lipid‐related lncRNAs and circRNAs to affect lipid metabolism reprogramming in cancer with distinct regulatory mechanisms. For *de novo* biogenesis of fatty acids, lncRNA *TINCR* and *FLJ22763* have been identified to modulate ACLY expression in different cancer cells. The first rate‐limiting enzyme in the *de novo* synthesis of fatty acid, ACC1, has been shown to be regulated by *circCAPRIN1*, lncRNAs *CTD‐2245E15.3* and *TSPEAR‐AS2*. With respect to other fatty acid synthetases, there are various lncRNAs and circRNAs involving the expression of these enzymes, functioning as the sponges of miRNAs, such as the lncRNA *SNHG25*/miR‐497‐5p/FASN axis, the *circFARSA*/miR‐330‐5p and miR‐326/FASN axis, the *circ_0 008078*/miR‐191‐5p/ELOVL4 axis, the *circ_0 008078*/miR‐191‐5p/ELOVL4 axis, the *linc00174*/miR‐145‐5p/SCD5 axis, and the *circ_0000073*/ miR‐1184/ FADS2 axis. For *de novo* biogenesis of cholesterols, lncRNAs *ZFAS1* and *AT102202* have been shown to regulate the expression of *HMGCR*, while *lnc30* and *circ_0000182* have been identified to modulate the expression of *SQLE*. Detailed mechanisms of these lipogenesis‐related lncRNAs and circRNAs in cancer are described in the main text.

**Figure 5:**
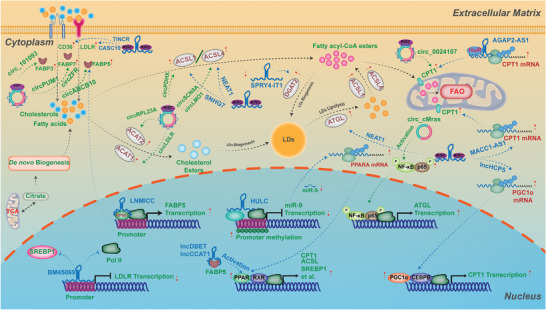
The lncRNAs & circRNAs in regulating lipid transport, lipid droplets (LDs) metabolism, and lipolysis of cancer. With respect to lipid transport, lipid droplet metabolism, and lipolysis in cancer, various lipid‐related lncRNAs and circRNAs have been shown to play variable roles through the RNA‐RNA, RNA‐protein, and RNA‐DNA interactions. For the RNA‐RNA interaction, lipid‐related lncRNAs and circRNAs act as sponges of miRNAs to release miRNAs‐mediated repression of target genes, such as the circ_ABCB10/miR‐620/FABP5 axis in nasopharyngeal carcinoma (NPC), the circ_101 093/FABP3/ FABP3 axis in lung adenocarcinoma (LUAD), and the lncHCP5/miR‐3619‐5p/CPT1 axis in gastric cancer. For the RNA‐protein interaction, these lipid‐related lncRNAs and circRNAs hold the potential to bind transcriptional factors, posttranslational modifiers, or RNA‐ binding proteins to modulate the expression, stability and activation of key rate‐limiting enzymes. For instance, *lncLNMICC* binds transcriptional factor‐NPM1 to promote the expression of *FABP5* in cervical cancer; lncRNA *CCAT1* binds USP49 to regulate FKBP51‐mediated AKT phosphorylation, thereby promoting *FABP5* expression in LUAD; lncRNA *AGAP2‐AS1* binds HuR protein to enhance protein stability of CPT1 in MSC‐cocultured Breast cancer (BC). For the RNA‐DNA interaction, lncRNA *BM450697* has been found to directly bind the DNA of the *LDLR* promoter, thereby inhibiting lipid uptake in hepatocellular carcinoma (HCC), while lncRNA *HULC* is able to induce methylation of CpG islands in the promoter of *miR‐9* to promote *ACSL1* expression and ACSL1‐mediated lipogenesis in HCC. Detailed mechanisms of these lncRNAs and circRNAs for lipid transport, LDs metabolism and lipolysis are described in the main text.

**Figure 6:**
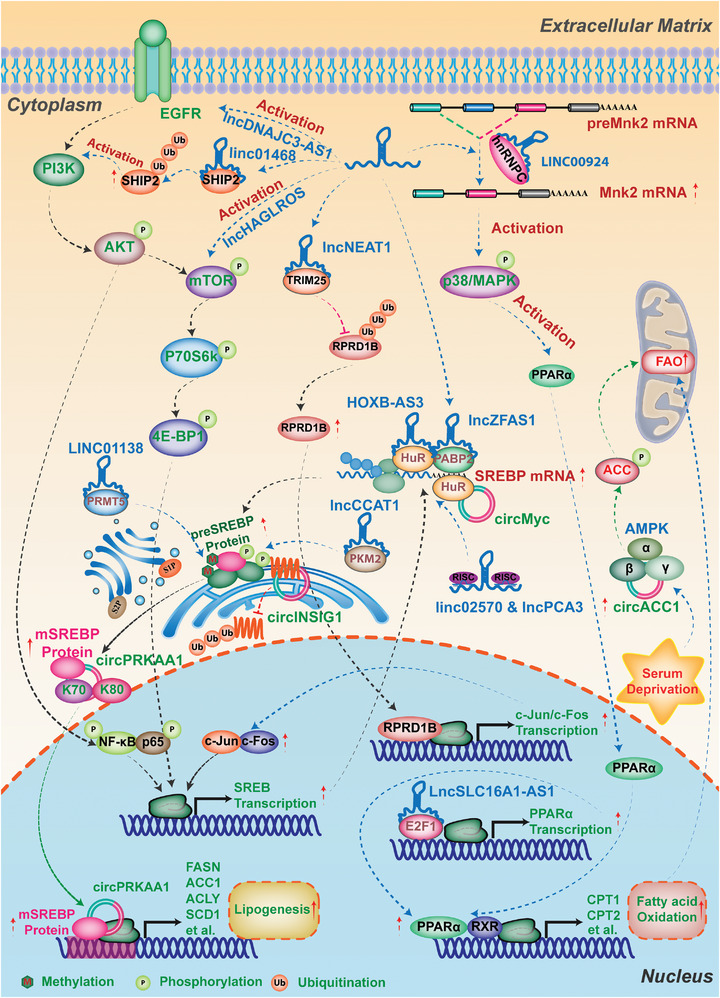
The lncRNAs & circRNAs in regulating lipid metabolic transcriptional factors and oncogenic signaling pathways of cancer. SREBP is the major transcriptional factor to regulate biogenesis of fatty acids and cholesterols at transcriptional levels. The lipid‐related lncRNAs and circRNAs in cancer can modulate the expression and stability of SREBPs through various regulatory mechanisms, including sponging miRNAs, binding protein and DNA, and affecting signaling pathways, even via modulating their regulators. Additionally, some lipid‐related lncRNAs and circRNAs have been shown to involve in regulating lipid metabolism in cancer via oncogenic signaling pathways, such as the PI3K‐AKT‐mTOR signaling pathway, the AKT/FoxO1/LXRα/RXR axis, and the p38 MAPK/PPARα signaling pathway. Detailed mechanisms of these lncRNAs and circRNAs in lipid metabolic transcriptional factors and oncogenic signaling pathways of cancer are described in the main text.

Apart from various key lipid metabolic enzymes, transcriptional factors and oncogenic signaling pathways as mentioned above, numerous studies have demonstrated that non‐coding RNAs, especially lncRNAs and circRNAs, are extensively dysregulated in various malignant tumors and play crucial roles in cancer metabolic reprogramming, especially in lipid metabolism. These dysregulated lncRNAs and circRNAs in lipid metabolic reprogramming (known as lipid‐metabolic related lncRNAs and circRNAs) not only support the demand of rapid ATP generation, biosynthesis of macromolecules and maintenance of appropriate redox status in cancer, but also promote cancer cells to disseminate to distal organs and resist to radiochemotherapy, even involving the mechanism of anti‐ferroptosis.^[^ ^12^, ^18^, ^101^, ^102^ ^]^ In the following sections, we will summarize the lipid‐metabolic related lncRNAs and circRNAs and their targets in lipid‐metabolic reprogramming of cancer, analyze their multiple regulatory mechanisms and display their various relevant biological functions in detail (**Figures** **4**, **5**, **6**, **Tables** **1**, **2**, **3**, **4**).

**Table 1:** The lncRNAs‐circRNAs in regulating de novo synthesis of fatty acid and cholesterol of cancer.

Lipid metabolic lncRNAs	Status in tumor	Lipid metabolic process	Cancer types	Clinical association	Functional impact	Interactor	Target/effect	Mechanistic classification	Refs
TINCR	Up‐regulated	De novo fatty acid synthesis	Nasopharyngeal carcinoma (NPC)	Overall survival↓ Disease free survival↓ Distant‐metastasis↑	Proliferation↑ Metastasis↑ Cisplatin resistance↑	ACLY	Increasing the cellular acetyl‐CoA level to promote lipid synthesis	Binding with protein (Binding ACLY protein to maintain its stability by inhibiting its ubiquitination‐mediated degradation)	[103]
FLJ22763	Down‐regulated	De novo fatty acid synthesis	Gastric cancer (GC)	Histological grade↑ Depth of invasion↑	Proliferation↑ Migration↑ Invasion↑ Tumorigenesis↑	N.A	mRNA and protein expression of ACLY↑	N.A	[104]
CTD‐2245E15.3	Up‐regulated	De novo fatty acid synthesis	Non‐small cell lung cancer (NSCLC)	N.A	Proliferation↑	ACC1	Promoting ACC1‐mediated lipogenesis to facilitate tumor cell proliferation	Binding with protein (Binding ACC1 to decrease phosphorylation of ACC1 at an inhibitory site)	[106]
TSPEAR‐AS2	Up‐regulated	De novo fatty acid synthesis	Colorectal cancer (CRC)	Predictor of overall survival	Fatty acid metabolism↑	N.A	Regulating ACC1 and FASN‐mediated the fatty acid synthesis	N.A	[107]
CircCAPRIN1	Up‐regulated	De novo fatty acid synthesis	Colorectal cancer (CRC)	TNM stages↑ Clinical stages↑ Overall survival↓ DFS↓	Proliferation↑ Migration↑ EMT↑	STAT2	Expression of ACC1↑ Fatty acid synthesis↑	Binding with protein (Binding to STAT2 to transcriptionally activate ACC0 gene expression)	[105]
SNHG25	Up‐regulated	De novo fatty acid synthesis	Endometrial cancer (EC)	Overall survival↓	Proliferation↑ Migration↑ Invasion↑	miR‐497‐5p	Regulating FASN‐mediated tumor growth	Sequestration of miRNAs (Releasing miR‐497‐5p‐mediated repression of FASN expression)	[108]
HAGLR	Up‐regulated	De novo fatty acid synthesis	Non‐small cell lung cancer (NSCLC)	Clinical stage↑ Lymph node metastasis status↑ Overall survival↓	Proliferation↑ Lipogenesis↑ Invasion↑	FASN	Regulating FASN‐mediated lipogenesis and growth	N.A	[116]
HOTAIR	Up‐regulated	De novo fatty acid synthesis	Nasopharyngeal carcinoma (NPC)	N.A	Proliferation↑ Lipogenesis↑ Invasion↑	N.A	Promoting FASN‐mediated lipogenesis, proliferation and invasion	N.A	[115]
PVT1	Up‐regulated	De novo fatty acid synthesis	Osteosarcoma (OS)	Metastasis status↑ Overall survival↓	Proliferation↑ Migration↑ Invasion↑ Cell cycle arrest↓ Apoptosis↓	miR‐195	Promoting FASN‐mediated lipogenesis, migration and invasion	Sequestration of miRNAs (Releasing miR‐195‐mediated repression of BCL2, CCND1 and FASN expression)	[109]
CircFARSA	Up‐regulated	De novo fatty acid synthesis	Non‐small cell lung cancer (NSCLC)	Overall survival↓	Proliferation↑ Lipogenesis↑ Invasion↑	miR‐330‐5p miR‐326	Promoting FASN‐mediated the proliferation and migration	Sequestration of miRNAs (Releasing miR‐330‐5p and miR‐326‐mediated repression of FASN expression)	[110]
CircWHSC1	Up‐regulated	De novo fatty acid synthesis	Breast cancer (BC)	Tumor stages↑ Lymphatic metastasis↑ Overall survival↓	Proliferation↑ Lipogenesis↑ Invasion↑	miR‐195‐5p	Promoting FASN‐mediated AMPK inhibition and mTOR activation to facilitate proliferation and migration	Sequestration of miRNAs (Releasing miR‐195‐5p‐mediated repression of FASN expression)	[111]
CircZFAND6	Up‐regulated	De novo fatty acid synthesis	Breast cancer (BC)	N.A	Proliferation↑ Metastasis↑	miR‐647	FASN‐mediated proliferation and metastasis↑	Sequestration of miRNAs (Releasing miR‐647‐mediated repression of FASN expression)	[112]
Circ_0018909	Up‐regulated	De novo fatty acid synthesis	Pancreatic carcinoma (PC)	Clinical stage↑	Proliferation↑ Migration↑ EMT↑ Apoptosis↓	miR‐545‐3p	FASN‐mediated progression↑ M2 macrophage polarization↑	Sequestration of miRNAs (Releasing miR‐545‐3p‐mediated repression of FASN expression)	[113]
CircMBOAT2 (hsa_circ_0 0 07334)	Up‐regulated	De novo fatty acid synthesis	Intrahepatic cholangiocarcinoma (ICC)	Tumor size↑ Overall survival↓	Proliferation↑ Colony formation↑ Cell cycle progression↑ Apoptosis↓	PTBP1	Stability of PTBP1↑ Lipid metabolism reprogramming↑	Binding with protein (Binding to PTBP1 to promote PTBP1‐mediated cytoplasmic export of FASN mRNA)	[114]
FASRL	Up‐regulated	De novo fatty acid synthesis	Hepatocellular carcinoma (HCC)	Overall survival↓	Proliferation↑ Migration↑ Apoptosis↓	ACACA	Decreasing the level of phosphorylated ACACA (Ser79)	Binding with protein (Binding ACACA to inhibit its phosphorylation, thus increasing FA synthesis)	[117]
Circ_0008078	Down‐regulated	De novo fatty acid biosynthesis‐Elongation	Esophagus cancer (EC)	Overall survival↓	Proliferation↑ Migration↑ Invasion↑ Tube formation↑	miR‐191‐5p	Promoting ELOVL4‐mediated proliferation and migration	Sequestration of miRNAs (Releasing miR‐191‐5p‐mediated repression of ELOVL4 expression)	[118]
Linc00174	Up‐regulated	Fatty acid desaturation	Thymic epithelial tumors (TETs)	Overall survival↓ Disease free survival↓ Distant‐metastasis↑	Proliferation↑ Migration↑ Lipogenesis↑	miR‐145‐5p	Expression of SCD5↑Lipogenesis and progression↑	Sequestration of miRNAs (Releasing miR‐145‐5p‐mediated repression of SCD5 expression)	[119]
UPAT	Up‐regulated	Fatty acid desaturation	Colorectal cancer (CRC)	N.A	Viability↑ Tumorigenicity↑	UHRF1	SCD1 and SPRY4 ‐mediated the survival and tumorigenicity↑	Binding with protein (Binding UHRF1 transcriptional factor to promote the expression of SPRY4 directly, SCD1 indirectly by interfering ubiquitination and degradation of UHRF1)	[120]
SNHG16	Up‐regulated	Fatty acid desaturation	Colorectal cancer (CRC)	Tumor stages↑	Proliferation↑ Migration↑ Lipid metabolism	26 unique miRNA families	Expression of many lipid‐metabolism related genes↑	Sequestration of miRNAs (Releasing half of 26 unique miRNA families‐mediated repression of SCD‐mediated lipid metabolism)	[121]
KDM4A‐AS1	Up‐regulated	Fatty acid desaturation	Esophageal squamous cell carcinoma (ESCC)	N.A	Viability↓ Migration↓	N.A	Stearoyl‐CoA desaturase↓ Fatty acid synthase expression↓ Reactive oxygen species ↑ Mitochondrial membrane potential ↓	KDM4AAS1‐encoded peptide inhibits the expression of SCD and FASN	[122]
Linc00460	Up‐regulated	Fatty acid desaturation	Osteosarcoma (OS)	Overall survival↓ Disease free survival↓ Distant metastasis↑	Proliferation↑ Migration↑ Invasion↑	miR‐1224‐5p	FADS1‐mediated proliferation and metastatic potential↑	Sequestration of miRNAs (Releasing miR‐1224‐5p‐mediated repression of FADS1 expression)	[123]
LINC01569	Up‐regulated	Fatty acid desaturation	Hypopharyngeal carcinoma (HPC)	N.A	Proliferation↑ Apoptosis↓	miR‐193a‐5p	FADS1‐mediated M2 macrophage polarization↑	Sequestration of miRNAs (Releasing miR‐193a‐5p‐mediated repression of FADS1 expression)	[124]
Circ_0000073	Up‐regulated	Fatty acid desaturation	Osteosarcoma (OS)	Clinical stage↑ Large tumor size↑ Metastasis↑ Overall survival↓	Proliferation↑ Metastasis↑	miR‐1184	FADS2‐mediated lipid synthesis↑	Sequestration of miRNAs (Releasing miR‐1184‐mediated repression of FADS2 expression)	[125]
ZFAS1	Up‐regulated	Cholesterol biogenesis	Pancreatic carcinoma (PC)	N.A	Proliferation↑ Invasion↑ Lipo‐metabolism↑	U2AF2	HMGCR mRNA Stability↑	Binding with protein (Binding U2AF2 to maintains HMGCR mRNA Stabilization)	[126]
AT102202	Down‐regulated	Cholesterol biosynthesis	Hepatocellular carcinoma (HCC)	N.A	**N.A**	N.A	HMGCR‐mediated cholesterol biosynthesis↑	N.A	[127]
lnc030	Up‐regulated	Cholesterol biosynthesis	Breast cancer stem cells (BCSCs)	Lymph node metastasis↑ Pathological grade↑ Overall survival↑	Tumor cell stemness↑ Tumorigenesis↑	PCBP2	Promoting SQLE‐mediated cholesterol synthesis to further active PI3K/Akt signaling	Binding with protein (Cooperating with poly(rC) binding protein 2(PCBP2) to stabilize squalene epoxidase (SQLE) mRNA)	[128]
Circ_0000182	Up‐regulated	De novo fatty acid synthesis	Gastric cancer (GC)	Tumor size↑	Cell proliferation↑	miR‐579‐3p	SQLE‐mediated cholesterol synthesis↑	Sequestration of miRNAs (Releasing miR‐579‐3p‐mediated repression of SQLE expression)	[129]

PS: **N.A**. Not Available.

**Table 2:** The lncRNAs‐circRNAs in regulating lipid transport and fatty acid oxidation of cancer.

Lipid metabolic lncRNAs	Status in tumor	Lipid metabolic process	Cancer types	Clinical association	Functional impact	Interactor	Target/effect	Mechanistic classification	Refs
TINCR	Down‐regulated	Lipid transport	Colorectal cancer (CRC)	N.A	Tumor progression↑	miR‐107	Mediating CD36‐mediated proliferation and apoptosis	Sequestration of miRNAs (Releasing miR‐107‐mediated repression of CD36 expression)	[130]
CASC19	Up‐regulated	Lipid transport	Non‐small cell lung cancer (NSCLC)	N.A	Proliferation↑ Metastasis↑	miR‐301b‐3p	Mediating LDLR‐mediated proliferation and metastasis	Sequestration of miRNAs (Releasing miR‐301b‐3p‐mediated repression of LDLR expression)	[131]
BM450697	Up‐regulated	Lipid transport	Hepatocellular carcinoma (HCC)	N.A	Lipid uptake↓	LDLR promoter	Mediating LDLR‐mediated LDL Uptake	DNA binding (Directly interacting with the DNA of the LDLR promoter to block interactions with Pol II and possibly SREBP1a at the promoter of LDLR)	[132]
CircRNA_101 093 (cir93)	Up‐regulated	Lipid transport	Lung adenocarcinoma (LUAD)	Ferroptosis resistance↑ Overall survival↓	Lipid peroxidation↓ Sensitive to ferroptosis↓	FABP3	Promoting FABP3‐mediated desensitizing ferroptosis	Binding with protein (Interacted with and increased FABP3, which transported AA to react with taurine in form of NAT)	[133]
Circ_ABCB10	Up‐regulated	Lipid transport	Glioma	N.A	Proliferation↑ Migration↑ Invasion↑	miR‐620	Promoting FABP5‐mediated the proliferation and migration	Sequestration of miRNAs (Releasing miR‐620‐mediated repression of FABP5 expression)	[134]
LNMICC	Up‐regulated	Lipid transport	Cervical cancer (CC)	Tumor size↑ Stromal invasion↑ Lymph node metastasis↑ Recurrence↑ Overall survival↓ Disease free survival↓	Proliferation↑ Lipogenesis↑ Metastasis↑ Lymphangiogenesis↑	NPM1	Promoting FABP5‐mediated lipogenesis and lymph node metastasis	Binding with protein (Directly binding transcriptional factor‐NPM1 to the promoter of the FABP5 gene, which mediated reprogramming of FA metabolism)	[135]
LncDBET	Up‐regulated	Lipid transport	Bladder cancer (BCa)	Tumor volume and weight↑	Proliferation↑ Migration↑ Viability↑ Apoptosis↓	FABP5	Activating FABP5‐mediated PPAR pathway	Binding with protein (METTL14 upregulates lncDBET by m6A modification, thereby activating the PPAR signaling pathway through direct interaction with FABP5)	[137]
CCAT1	Up‐regulated	Lipid transport	Lung adenocarcinoma (LUAD)	Histology grade↑ Overall survival↓ Disease free survival↓	Lipogenesis↑ Proliferation↑ Angiogenesis↑	FABP5, USP49, RAPTOR	Activating PPAR‐RXR transcriptional complex and mTOR pathway	Binding with protein (Binding FABP5 to translocate FA into nuclear to facilitate PPAR/RXR transcriptional complex, and binding USP49 to regulate FKBP51‐mediated AKT phosphorylation, but also binds RAPTOR to stabilize PI3K pathway)	[138]
FAM201A	Down‐regulated	Lipid transport	Neuroblastoma (NB)	N.A	Proliferation↑ Migration↑ Invasion↑	FABP5	Expression of FABP5↑ FABP5‐medicated MAPK signaling pathway↑	Encoding peptides and binding with protein (Encoding peptides‐NBASP reduced the expression of FABP5 via the ubiquitin proteasome pathway)	[136]
Circ_ZFR	Up‐regulated	Lipid transport	Breast cancer (BC)	N.A	Proliferation↑ Migration↑ Invasion↑ EMT↑	miR‐223‐3p	Promoting FABP7‐mediated tumor progression	Sequestration of miRNAs (Releasing miR‐223‐3p‐mediated repression of FABP7 expression)	[140]
CircPUM1	Up‐regulated	Lipid transport	Clear cell renal cell carcinoma (ccRCC)	N.A	Proliferation↑ Migration↑ Invasion↑	miR‐340‐5p	Promoting FABP7‐mediated tumor proliferation	Sequestration of miRNAs (Releasing miR‐340‐5p‐mediated repression of FABP7 expression)	[139]
AGAP2‐AS1	Up‐regulated	Fatty acid oxidation (FAO)	MSC‐cocultured Breast cancer (BC)	Predictor of trastuzumab response	Tumor cell stemness↑ Trastuzumab resistance↑	HuR miR‐15a‐5p	Promote CPT1‐mediated FAO to facilitated the stemness and trastuzumab resistance	Binding with protein (Binding HuR protein to enhance protein stability of CPT1) Sequestration of miRNAs (Releasing miR‐15a‐5p‐mediated repression of CPT1 expression)	[141]
lncHCP5	Up‐regulated	Fatty acid oxidation (FAO)	Gastric cancer (GC)	N.A	Tumor cell stemness↑ Chemoresistance↑	miR‐3619‐5p	Regulating PGC1α‐CPT1‐mediated FAO to facilitate stemness and chemo‐resistance	Sequestration of miRNAs (Releasing miR‐3619‐5p‐mediated repression of PPARGC1A/ PGC1α expression and facilitating PGC1α interacted with CEBPB to induce CPT1 transcription)	[142]
Circ_0024107	Up‐regulated	Fatty acid oxidation (FAO)	Gastric cancer (GC)	TNM stages↑ Lymphatic metastasis↑ Overall survival↓	Migration↑ Invasion↑	miR‐5572 miR‐6855‐5p	CPT1A‐mediated fatty acid oxidation↑	Sequestration of miRNAs (Releasing miR‐5572 and miR‐6855‐5p‐mediated repression of CPT1A expression)	[144]
MACC1‐AS1	Up‐regulated	Fatty acid oxidation (FAO)	Gastric cancer (GC)	N.A	Tumor cell stemness↑ Chemoresistance↑ Proliferation↑	miR‐145‐5p	Increasing the expression of FAO enzyme to regulate stemness and chemoresistance	Sequestration of miRNAs (Releasing miR‐145‐5p‐mediated repression of stemness genes and FAO enzyme expression levels)	[143]
SOCS2‐AS1	Up‐regulated	Fatty acid oxidation (FAO)	Thyroid cancer (TC)	Overall survival↓	Proliferation↑ Progression↑	P53	P53 protein degradation↑ Fatty acid oxidation↑	Binding with protein (Binding P53 to regulate its protein turnover)	[145]

PS: **N.A**. Not Available.

**Table 3:** The lncRNAs‐circRNAs in regulating the esterification of lipids and the metabolism of lipid droplets (LDs) in cancer.

Lipid metabolic lncRNAs	Status in tumor	Lipid metabolic process	Cancer types	Clinical association	Functional impact	Interactor	Target/effect	Mechanistic classification	Refs
HULC	Up‐regulated	Long‐chain fatty acids esterification	Hepatocellular carcinoma (HCC)	N.A	Proliferation↑ Lipid metabolism↑	miR‐9	Regulating HCC growth through ACSL1‐mediated lipogenesis	Epigenetic modification of miRNAs gene (Releasing miR‐9‐mediated repression of ACSL1 expression through inducing methylation of CpG islands in the promoter of miR‐9)	^[^ ^148^ ^]^
CircPDHX	Up‐regulated	Long‐chain fatty acids esterification	Prostate cancer (PCa)	N.A	Proliferation↑ Migration↑ Fatty acid metabolism↑	miR‐497‐5p	Promoting ACSL1‐mediated proliferation and migration	Sequestration of miRNAs (Releasing miR‐497‐5p‐mediated repression of ACSL1 expression)	^[^ ^150^ ^]^
SNHG7	Up‐regulated	Long‐chain fatty acids esterification	Thyroid cancer (TC)	N.A	Proliferation↑ Migration↑	miR‐449a	Promoting ACSL1‐mediated proliferation and migration	Sequestration of miRNAs (Releasing miR‐449a‐mediated repression of ACSL1 expression)	^[^ ^151^ ^]^
PRADX	Up‐regulated	Long‐chain fatty acids esterification	Glioblastoma (GBM)	Poor prognosis↑	Energy metabolism↑ Tumorigenesis↑	EZH2	Promoting ACSL1‐mediated basal respiration, proton leak and ATP production	Binding with protein (Binding EZH2 to recruit H3K27me3 of BLCAP promoter and further to activate the phosphorylation of STAT3 and its downstream genes’ expression, including ACSL1)	^[^ ^149^ ^]^
CBSLR	Up‐regulated	Long‐chain fatty acids esterification	Gastric cancer (GC)	TNM stage↑ Overall survival↓ Disease free survival↓	Ferroptosis↓ Chemoresistance↑	YTHDF2	Inhibiting ACSL4‐mediated ferroptosis under hypoxia	Binding with protein (Binding YTHDF2 to form a CBSLR/ YTHDF2/CBS signaling axis to decrease the stability of CBS mRNA, which further reduces the methylation of the ACSL4 protein, leading to protein polyubiquitination and degradation of ACSL4.)	^[^ ^155^ ^]^
CircLMO1	Down‐regulated	Long‐chain fatty acids esterification	Cervical cancer	FIGO staging↑	Proliferation↑ Metastasis↑	miR‐4192	Promoting ACSL4‐mediated ferroptosis	Sequestration of miRNAs (Releasing miR‐4192‐mediated repression of ACSL4 expression)	^[^ ^153^ ^]^
CircSCN8A	Down‐regulated	Long‐chain fatty acids esterification	Non‐small cell lung cancer (NSCLC	Aggressive clinicopathological characteristics↑ Poor prognosis↑	Proliferation↑ Migration↑ Invasion↑ EMT↑	miR‐1290	Promoting ACSL4‐mediated ferroptosis	Sequestration of miRNAs (Releasing miR‐1290‐mediated repression of ACSL4 expression)	^[^ ^154^ ^]^
NEAT1	Up‐regulated	Long‐chain fatty acids esterification	Prostate cancer (PCa)	N.A	Proliferation↑ Invasion↑ Docetaxel resistance↑	miR‐34a‐5p miR‐204‐5p	Regulating ACSL4‐mediated docetaxel resistance	Sequestration of miRNAs (Releasing miR‐34a‐5p and miR‐204‐5p‐mediated repression of ACSL4 expression)	^[^ ^152^ ^]^
CircLDLR (circ_0 0 06877)	Up‐regulated	Cholesterol esterification	Colorectal cancer (CRC)	Poor prognosis↑	Proliferation↑ Migration↑ Invasion↑ Cholesterol levels↑	miR‐30a‐3p	Promoting SOAT1‐mediated cell growth, migration, invasion and cholesterol levels increase	Sequestration of miRNAs (Releasing miR‐30a‐3p‐mediated repression of SOAT1 expression)	^[^ ^156^ ^]^
CircRPL23A	Down‐regulated	Cholesterol esterification	Clear cell renal cell carcinoma (ccRCC)	N.A	Proliferation↑ Migration↑ Invasion↑ Apoptosis↓	miR‐1233	Inhibiting ACAT2‐mediated suppressing cell cycle progression, proliferation, migration and invasion	Sequestration of miRNAs (Releasing miR‐1233‐mediated repression of ACAT2 expression)	^[^ ^157^ ^]^
Linc01410	Up‐regulated	Lipid Drops formation	Cervical cancer	Poor overall survival↑	Lipid Drop accumulation↑ Invasion↑ Migration↑	miR‐532‐5p	Increasing FASN and PLIN2 expression to facilitate the LD accumulation	Sequestration of miRNAs (Releasing miR‐532‐5p‐mediated repression of FASN expression)	^[^ ^158^ ^]^
SPRY4‐IT1	Up‐regulated	Lipid Drops formation	Melanoma	N.A	Lipin 2‐mediated lipid metabolism↑ Apoptosis↓	lipin2	Accumulation of lipin2 protein↓ Expression of DGAT2↓	Binding with protein (Binding lipin2 to decrease its accumulation)	^[^ ^159^ ^]^
NEAT1	Up‐regulated	Lipolysis	Hepatocellular carcinoma (HCC)	Tumor size↑ DAG and FFA levels↑ Overall survival↓	Proliferation↑ Lipolysis↑	miR‐124‐3p	ATGL‐mediated Lipolysis↑ FAO↑	Sequestration of miRNAs (Releasing miR‐124‐3p‐mediated repression of ATGL expression)	^[^ ^160^ ^]^
NEAT1	Up‐regulated	Lipolysis	Ovarian cancer	N.A	Proliferation↑ Migration↑ Invasion↑	Let‐7 g	Promoting ATGL‐mediated lipolysis and tumor progression	Sequestration of miRNAs (Releasing let‐7 g‐mediated repression of MEST expression)	^[^ ^197^ ^]^
Circ‐cMras	Down‐regulated	Lipolysis	Lung adenocarcinoma (LAC)	N.A	Progression↑ Tumorigenesis↑	N.A	Modulating ABHD5/ATGL axis mediated in the proliferation and aggression	Signaling pathway (Regulating ABHD5 possibly via NF‐κB signaling pathway)	^[^ ^161^ ^]^
CircRIC8B	Up‐regulated	Lipolysis	Chronic lymphocytic leukemia (CLL)	Advanced progression ↑ Poor prognosis↑	Proliferation↑ Lipid accumulation↑	miR‐199b‐5p	Promoting LPL ‐mediated lipid metabolism alteration and the progression	Sequestration of miRNAs (Releasing miR‐199b‐5p‐mediated repression of LPL expression)	^[^ ^162^ ^]^
NMRAL2P	Down‐regulated	Lipolysis	Gastric cancer (GC)	N.A	Proliferation↑ Migration↑ Invasion↑ Cell cycle progression↑ Apoptosis↓	N.A	Inhibiting ACOT7‐mediated hydrolysis of fatty acyl‐CoAs	Epigenetic modification (Down‐regulation of ACOT7 through inducing methylation of CpG islands in its promoter by interacting DNMT3b)	^[^ ^163^ ^]^
COL18A1‐AS1	Down‐regulated	lipid browning	Clear cell renal cell carcinoma (ccRCC)	Overall survival↓ Tumor grade and stage↑	Tumor progression↑ Lipid browning and consumption↑	miR‐1286	Increasing the expression of KLF12 to regulate UCP1‐mediated lipid browning	Sequestration of miRNAs (Releasing miR‐1286‐mediated repression of KLF12 expression)	^[^ ^164^ ^]^

PS: **N.A**. Not Available.

**Table 4:** The lncRNAs‐circRNAs in regulating lipid metabolic transcriptional factors, oncogenic signaling pathways of lipid metabolism in cancer.

Lipid metabolic lncRNAs&circRNA	Status in tumor	Lipid metabolic process	Cancer types	Clinical association	Functional impact	Interactor	Target/effect	Mechanistic classification	Refs
Linc02570	Up‐regulated	Transcriptional factors	Nasopharyngeal carcinoma (NPC)	Histology grade↑ Disease free survival↓	Proliferation↑ Migration↑ Invasion↑ Viability↑ Lipogenesis↑	miR‐4649‐3p	Increasing SREBP1 and FASN expression to facilitate lipid metabolism	Sequestration of miRNAs (Releasing miR‐4649‐3p‐mediated repression of SREBP1 and FASN expression)	[165]
PCA3	Up‐regulated	Transcriptional factors	Prostate cancer (PCa)	N.A	Proliferations↑ Lipid metabolic disorders↑	miR‐132‐3p	Increasing the expression of SREBP1 to regulate the cellular triglyceride and cholesterol levels	Sequestration of miRNAs (Releasing miR‐132‐3P‐mediated repression of SREBP1 expression)	[166]
SNHG16	Up‐regulated	Transcriptional factors	Pancreatic carcinoma (PC)	N.A	Proliferation↑ Migration↑ Invasion↑ Lipid metabolism↑	miR‐195	Increasing the expression of SREBP2 to regulate lipogenesis and progression	Sequestration of miRNAs (Releasing miR‐195‐mediated repression of SREBP2 expression)	[167]
ZFAS1	Up‐regulated	Transcriptional factors	Colorectal cancer (CRC)	Tumor size↑ Invasive status↑ microsatellite stability↓	Proliferation↑ Enrichment of lipids↑ Invasion↑	PABP2	Increase SREBP1, FASN, and SCD1 expression to facilitate the accumulation of lipids	Binding with protein (Cooperating with polyadenylate binding protein 2 to maintain SREBP1 mRNA stability)	[171]
Linc01138	Up‐regulated	Transcriptional factors	Clear cell renal cell carcinoma (ccRCC)	TNM stage↑ Overall survival↓	Proliferation↑ Lipid metabolism↑	PRMT5	Regulating tumor growth through SREBP1‐mediated lipid desaturation	Binding with protein ( Binding PRMT5 protein to enhance protein stability of SREBP1)	[172]
CCAT1	Up‐regulated	Transcriptional factors	Osteosarcoma (OS)	N.A	Proliferation↑ Warburg effects↑ Lipogenesis↑	PKM2	Upregulating the phosphorylation of SREBP2 to promote lipogenesis	Binding with protein (Binding PKM2 to phosphorylate SREBP2 at T610 which affected the stability of SPREBP2)	[173]
CircPRKAA1	Up‐regulated	Transcriptional factors	Hepatocellular carcinoma (HCC)	N.A	Proliferation↑ Clonogenicity↑	Ku80/Ku70	Promoting mSREBP1‐mediated increasing fatty acid synthesis to promote cancer growth	Protein and DNA binding (Stabilizing mSREBP‐1 and enhancing its transcriptional activity through interaction with Ku proteins and selectively binding to the promoters of the ACC1, CLY, SCD1, and FASN genes to recruit mSREBP‐1)	[174]
HOXB‐AS3	Up‐regulated	Transcriptional factors	Endometrial cancer (EC)	Diagnostic indicator	Proliferation↑ Invasion↑ Apoptosis↓	PTBP1	Expression of SREBP1 and its downstream SCD1 and phosphorylated ACLY Ser455↑	Binding with protein (Binding to PTBP1 to form an RNA protein complex, promoting the expression of SREBP1)	[168]
CircMyc (hsa_circ_00 85533)	Up‐regulated	Transcriptional factors	Breast cancer (BC)	Tumor volume↑ Clinical stages↑ Lymphatic metastasis↑	Proliferation↑ Invasion↑	HuR Myc	SREBP1mRNA stability and transcription ↑ SREBP1‐mediated lipogenesis↑	Binding with protein (Binding HuR to regulate SREBP1 mRNA stability; Binding Myc to facilitate transcription of SREBP1)	[169]
CircREOS	Down‐regulated	Transcriptional factors	Osteosarcoma (OS)	N.A	Proliferation↓ Invasion↓	HuR	MYC‐medicated FASN activation↓	Binding with protein (Binding HuR to degrade MYC mRNA, further reducing ACC1 activation)	[170]
LINC00326	Down‐regulated	Transcriptional factors	Hepatocellular carcinoma (HCC)	N.A	Proliferation↓	CCT3	EGR1, CYR61 and GLIPR1‐mediated lipogenesis↓ lipolysis↑	Binding with protein (Binding CCT3 to impede CCT3's confinement of CREM, CREB and ATF)	[176]
CircINSIG1	Up‐regulated	Transcriptional factors	Colorectal cancer (CRC)	TNM stages↑ Clinical stages↑ Overall survival↓	Proliferation↑ Metastasis↑	INSIG1	Cholesterol biosynthesis↑	Encoding peptides and binding with protein (Encoding peptides‐ circINSIG1‐121 to promote INSIG1 degradation via recruiting CUL5‐ASB6 complex, further inducing nSREBP2 increase)	[175]
DNAJC3‐AS1	Up‐regulated	Signaling Pathways	Colorectal cancer (CRC)	Local invasion↑ Clinical stages↑ Overall survival↓	Proliferation↑ Migration↑ Invasion↑ Lipid accumulation↑	N.A	Regulating ACC1 and FASN‐mediated the progression	Modulating signaling pathways (Regulating the Expression of ACC1/FASN Via EGFR/PI3K/AKT/NF‐Kb/SREBP1 Signaling Pathway)	[177]
HAGLROS	Up‐regulated	Signaling Pathways	Intrahepatic cholangiocarcinoma (ICC)	Tumor stage↑ Differentiation↓ Overall survival↓	Proliferation↑ Migration↑ Lipid accumulation↑	mTOR signaling pathway	Activation of mTOR pathway can increase lipid metabolism‐related proteins expression and PPARγ	N.A	[178]
Linc01468	Up‐regulated	Signaling Pathways	Non‐alcoholic fatty liver disease (NAFLD) associated hepatocellular carcinoma (HCC)	Hemoglobin A1C (HbA1C), triglyceride (TG), total cholesterol (TC), cirrhosis levels↑ Tumor size↑ Tumor stage↑ TNM stage↑ Microvascular invasion↑ Overall survival↓	Chemoresistance↑ Tumorigenesis↑	SHIP2	Activation of Akt/mTOR signaling pathway mediated increase of intracellular TG and TC	Binding with protein (Binding SHIP2 protein to induce CUL4A by promoting the ubiquitinated degradation of SHIP2)	[179]
lncARSR	Up‐regulated	Signaling Pathways	Hepatocellular carcinoma (HCC) and Nonalcoholic fatty liver disease (NAFLD)	N.A	Proliferation↑ Migration↑ Lipid accumulation↑	YAP1	Activating IRS2/ AKT pathway to increase adipogenesis related proteins (FASN, SCD1 and GPA) and PPARγ	Binding with protein (Binding YAP1 protein to maintain its nuclear translocation to activate IRS2/ AKT pathway)	[180]
NEAT1	Up‐regulated	Signaling Pathways	Gastric cancer (GC)	Overall survival↓	Lymphatic metastasis↑	TRIM25	Regulating RPRD1B/c‐Jun/c‐Fos/ SREBP1 axis‐mediated lipogenesis and lymph node metastasis	Binding with protein (Binding TRIM25 to reduce the degradation of RPRD1B protein, which transcriptionally upregulates c‐Jun/c‐Fos and activate the c‐Jun/c‐Fos/SREBP1 axis)	[181]
lncHR1	Down‐regulated	Signaling Pathways	Hepatocellular carcinoma (HCC)	N.A	N.A	N.A	SREBP‐1c‐mediated the fatty acid synthesis↑	Modulating signaling pathways (Suppressing the phosphorylation of DK1/AKT/FoxO1 axis to weaken the combinatorial capacity of LXRα/RXR binding to LXREs to decrease the expression of SREBP‐1c)	[182]
SLC16A1‐AS1	Up‐regulated	Signaling Pathways	Bladder cancer (BCa)	N.A	Invasion↑ Metabolic reprogramming (increasing aerobic glycolysis, mitochondrial respiration and FAO) ↑	E2F1	Regulating PPARA‐mediated FAO to promote cancer invasiveness	Binding with protein (Binding E2F1 transcriptional factor to promote the expression of PPARA, a key mediator of fatty acid β‐oxidation)	[183]
Linc00924	Up‐regulated	Signaling Pathways	Peritoneal metastasis associated gastric cancer (GC)	Lymph node status↑ TNM stage↑ Overall survival↓ Disease free survival↓	Invasion↑ Metastasis↑	hnRNPC	Activation of p38/PPARα signaling pathway mediated FAO and FA uptake	Binding with protein (Binding hnRNPC to regulate the alternative splicing of Mnk2 pre‐mRNA, thereby decreasing Mnk2a splicing and activating the p38 MAPK/PPARα signaling pathway)	[184]
CircACC1 (circ001391)	Up‐regulated	Signaling Pathways	Colorectal cancer (CRC)	N.A	FAO↑ Glycolysis↑	AMPK β and γ Subunits	Promoting AMPK‐mediated in inhibition of fatty acid synthesis (FAS) and stimulation of FAO via the phosphorylation of ACC1 and ACC‐2, respectively.	Binding with protein (Binding AMPK β and γ Subunits to facilitate AMPK Holoenzyme assembly, stability, and activity)	[185]
EcircCUX1 (circ30402)	Up‐regulated	Signaling Pathways	Neuroblastoma (NB)	Poor differentiation↑ Higher mitosis karyorrhexis index↑ Advanced international neuroblastoma staging system (INSS) stages↑ Overall survival↓	Proliferation↑ Tumorigenesis↑ Aggressiveness↑ Fatty acid oxidation↑ Mitochondrial activity↑	ZRF1 BRD4	Promoting ALDH3A1, NDUFA1, and NDUFAF5‐mediated lipid metabolic reprogramming and mitochondrial complex I activity	Encoding peptides and Protein biding (p113‐encoded by circCUX1, interacts with ZRF1 and BRD4 to form a transcriptional regulatory complex, facilitating ALDH3A1, NDUFA1, and NDUFAF5 transcription)	[186]

PS: **N.A**. Not Available.

### 4.1 The lncRNAs‐circRNAs in Regulating de Novo Synthesis of Fatty Acid and Cholesterol of Cancer

In the initial step of *de novo* lipogenesis, ACLY is responsible for acetyl‐CoA synthesis, which provides the primitive fuels for both fatty acid and cholesterol synthesis pathways (Figure 4 and Table 1). A novel lipid metabolic related lncRNA, *TINCR*, was identified to be significantly overexpressed in nasopharyngeal carcinoma (NPC), and can promote proliferation, metastasis and cisplatin resistance of NPC by affecting ACLY‐mediated *de novo* lipid biosynthesis. Mechanistically, *TINCR* interacts with ACLY directly and inhibited its ubiquitin degradation to increase cellular total acetyl‐CoA levels for lipid synthesis and histone acetylation.^[^ ^103^ ^]^ Moreover, *lncRNA FLJ22763*, which is downregulated in gastric cancer (GC), negatively regulates the mRNA and protein expression of ACLY to suppress the proliferation, migration, and invasion of GC cells as a tumor suppressor gene.^[^ ^104^ ^]^

As the acetyl‐CoA is increasingly synthesized, it is carboxylated to form malonyl‐CoA by ACC1, which is a rate‐limiting enzyme in the *de novo* synthesis of fatty acid. The lipid‐metabolic related lncRNAs and circRNAs in cancers have been found to regulate the expression of ACC1 mainly through modulating its mRNA transcription and activation of protein phosphorylation (Figure 4). For instance, *circCAPRIN1* is upregulated in colorectal cancer (CRC) as an oncogenic regulator. It can directly bind transcriptional factor STAT2 to facilitate the transcription of ACC1, which further promotes adipogenesis, proliferation, metastasis, and EMT of CRC.^[^ ^105^ ^]^ Additionally, *lncRNA CTD‐2245E15.3*, upregulated in non‐small cell lung cancer (NSCLC), promotes fatty acid biosynthesis of cancer cells mainly by interacting with ACC1 to promote its activity by reducing phosphorylation of an inhibitory site of ACC1.^[^ ^106^ ^]^ Another lncRNA, *TSPEAR‐AS2*, is identified to regulate ACC1 and FASN‐mediated fatty acid synthesis of CRC through Gene Set Variation Analysis (GSVA) and the construction of a prognostic signature with a ceRNA network, which is closely associated with the overall survival of CRC. However, the detailed mechanisms of how *TSPEAR‐AS2* regulates ACC1 and FASN expression require to be further explored.^[^ ^107^ ^]^

FASN is responsible for the consecutive condensation of malonyl‐CoA and acetyl‐CoA to form long‐chain saturated fatty acids, mostly 16‐carbon palmitate. Recently, the dysregulated lipidmetabolic‐related lncRNAs and circRNAs in cancers have been demonstrated to play key roles in regulating FASN expression by the ceRNA mechanism (Figure 4). For instance, *lncRNA SNHG25*, upregulated in endometrial cancer, was identified to regulate *FASN* expression by inhibiting miR‐497‐5p‐mediated repression.^[^ ^108^ ^]^ Other lipid‐metabolic related lncRNAs and circRNAs of cancers have also been identified to regulate *FASN* expression in the same ways, such as the *PVT1/*miR‐195*/FASN* axis in osteosarcoma (OS),^[^ ^109^ ^]^ the *circFARSA/*miR‐330‐5p and miR‐326*/FASN* axis in NSCLC,^[^ ^110^ ^]^ the *circWHSC1*/miR‐195‐5p*/FASN* and *circZFAND6*/ miR‐647*/FASN* axis in breast cancer (BC),^[^ ^111^, ^112^ ^]^ and the *circ_00 18 909/miR‐545‐3p/FASN* axis in pancreatic carcinoma (PC).^[^ ^113^ ^]^ Even *FASN* mRNA transportation from the nucleus to the cytoplasm has been shown to be mediated by *circMBOAT2*, which facilitates the lipid metabolic profile and redox homeostasis in intrahepatic cholangiocarcinoma (ICC).^[^ ^114^ ^]^ Additionally, lncRNA *HOTAIR* and *HAGLR*, overexpressed in NSCLC and NPC, respectively, have been shown to regulate *FASN* expression and be involved in FASN‐mediated lipogenesis, proliferation, and invasion of cancer. However, the mechanisms of these two lncRNAs in regulating *FASN* expression need to be further explored in detail.^[^ ^115^, ^116^ ^]^ Recently, a novel lncRNA *FASRL* has been identified to promote the proliferation and metastasis of hepatocellular carcinoma (HCC) via directly binding ACACA (acetyl‐CoA carboxylase 1) to facilitate *de novo* synthesis of fatty acid.^[^ ^117^ ^]^

To replenish the cellular pool of non‐essential FAs, such as monounsaturated 18‐carbon FA oleate (18:1) or 16‐carbon palmitoleate (16:1), *de novo* palmitate needs to undergo further elongation by ELOVL and SCD or fatty acid desaturases (FADS). The dysregulated lipid‐metabolic related lncRNAs and circRNAs in cancers can also play crucial roles in regulating elongation and desaturation of *de novo* fatty acids (Figure 4). For the elongation of de novo fatty acids, *circ_0 0 08078* was identified to be downregulated in esophagus cancer (EC), which promoted *ELOVL4* expression via miR‐191‐5p, and regulated the proliferation, tube formation, migration and invasion of EC.^[^ ^118^ ^]^ For the desaturation of *de novo* fatty acids, these lipid‐metabolic related lncRNAs and circRNAs can involve the SCD‐mediated desaturation of *de novo* fatty acids. For instance, a novel *linc00174* has been identified to modulate the SCD5‐meditated lipid production by absorbing miR‐145‐5p in thymic epithelial tumors (TETs).^[^ ^119^ ^]^ Moreover, *lncRNA UPAT* can regulate SCD1 indirectly to improve the tumorigenicity of CRC cells by interacting with and interfering with UHRF1's ubiquitination.^[^ ^120^ ^]^ Additionally, through RNA sequencing and mRNA/ncRNA profiling screen, lncRNA *SNHG16* is discovered to be significantly upregulated in early phase of CRC and holds AGO/miRNA target sites with several miRNA families, half of which targeted the 3′‐UTR of SCD with high confidence.^[^ ^121^ ^]^ Recently, a novel peptide encoded by *KDM4A‐AS1* has been shown to repress the expression of SCD and other fatty acid synthase to weaken the viability and migratory capacity of esophageal squamous cell carcinoma (ESCC), in result of increasing the reactive oxygen species level and breaking mitochondrial redox homeostasis.^[^ ^122^ ^]^ On the other hand, some lipid‐metabolic related lncRNAs and circRNAs in cancer can also regulate the FADS‐mediated desaturation of de novo fatty acids, apart from SCD. For example, *linc00460* has been identified to be upregulated in osteosarcoma (OS), which functions as a sponge of miR‐1224‐5p to promote OS progression by upregulating FADS1 expression.^[^ ^123^ ^]^ *LINC01569* has been identified to enrich in hypopharyngeal carcinoma associated macrophages to promote its M2 polarization via releasing miR‐193a‐5p‐mediated repression of *FADS1* expression. These polarized M2 macrophages further accelerate the progression of hypopharyngeal carcinoma.^[^ ^124^ ^]^ Recently, *Circ_0000073* has been shown to regulate the lipid synthesis of osteosarcoma in the same way of promoting the expression of *FADS2* as a sponge of *miR‐1184*.^[^ ^125^ ^]^

In the process of *de novo* cholesterol biosynthesis, HMGCR is one of the rate‐limiting enzymes that can be regulated by dysregulated lipid‐metabolic related lncRNAs and circRNAs in cancers (Figure 4). For instance, when overexpressed in pancreatic carcinoma (PC), *lncRNA ZFAS1* can stabilize and increase HMGCR mRNA to promote lipid accumulation in PC by binding to U2AF2, one component of spliceosomes.^[^ ^126^ ^]^ Moreover, a bioinformatic analysis of the roles of lncRNA in facilitating epigallocatechin‑3‑gallate (EGCG) on cholesterol metabolism identified *lncRNA AT102202* with potential to target HMGCR mRNA for regulating cholesterol metabolism of HepG2 cells. However, its mechanism needs to be further explored in the future.^[^ ^127^ ^]^ Additionally, squalene epoxidase is another key rate‐limiting enzyme in cholesterol biosynthesis. Recently, *lnc030* has been shown to promote cholesterol synthesis in breast cancer stem cells (BCSCs) by cooperating with poly(rC) binding protein 2 (PCBP2) to stabilize SQLE mRNA, which leads to increased cholesterol production. This increased cholesterol, in turn, activates the PI3K/Akt signaling pathway and promotes the stemness of BCSCs.^[^ ^128^ ^]^ *Circ_0000182* has been found to facilitate the proliferation and cholesterol synthesis in cholesterol synthesis in via releasing miR‐579‐3p‐mediated repression of *SQLE* expression.^[^ ^129^ ^]^

### 4.2 The lncRNAs‐circRNAs in Regulating Lipid Transport and Fatty Acid Oxidation of Cancer

Exogenous lipids enter into the cancer cells mainly through two known lipid transporters in the plasma membrane, CD36 or LDLR. The dysregulated lipid‐metabolic related lncRNAs and circRNAs in cancers can modulate extracellular lipids uptake by regulating these transporters (Figure 5 and Table 2). For instance, lncRNA *TINCR* has been found to be downregulated in CRC cells, but it can relieve the *miR‐107*‐mediated repression of CD36 expression, promoting apoptosis and inhibiting the proliferation of CRC cells.^[^ ^130^ ^]^ Similarly, overexpressed lncRNA CASC19 in NSCLC can positively regulate LDLR by targeting miR‐301b‐3p to facilitate proliferation and metastasis of NSCLC cells. Additionally, some lipid‐metabolic related lncRNAs and circRNAs in cancer can modulate LDLR expression epigenetically.^[^ ^131^ ^]^ For example, as an antisense RNA that overlaps the promoter of the LDLR gene, *lncRNA BM450697* can decrease the expression of LDLR by inhibiting interaction between RNA polymerase II (Pol II) or transcription factor SREBP1a and the promoter of LDLR in HCC.^[^ ^132^ ^]^

The role of fatty acid‐binding proteins (FABPs) in lipid metabolism is to transport newly synthesized or extracellular fatty acids for energy supply and storage. The isoforms of FABPs have been upregulated to facilitate viability, proliferation, migration and metastasis in various cancers, in which the dysregulated lipid‐metabolic related lncRNAs and circRNAs in cancer can also play significantly important roles (Figure 5). For instance, *circRNA_101 093*, derived from lung adenocarcinoma (LUAD) patients’ plasma exosome, has been identified to interact and increase FABP3, which then transports arachidonic acid and desensitized LUAD cells to ferroptosis.^[^ ^133^ ^]^ Moreover, the dysregulated lipid‐metabolic related lncRNAs and circRNAs can regulate the expression of FABP5 in various cancer with different mechanisms: On the one hand, *circ‐ABCB10* can promote FABP5‐mediated the proliferation and migration of glioma by absorbing miR‐620.^[^ ^134^ ^]^ Moreover, *LncLNMICC* can bind transcriptional factor‐NPM1 to the promoter of the FABP5 gene to facilitate FABP5‐mediated lipogenesis, lymph node metastasis of cervical cancer (CC).^[^ ^135^ ^]^ Neuroblastoma‐associated small protein (NBASP) encoded by lncRNA *FAM201A* has recently been found to inhibit the expression of *FABP5* via the ubiquitin proteasome pathway in neuroblastoma, which further repressed the progression of neuroblastoma by inactivating the MAPK pathway mediated by downregulating *FABP5*.^[^ ^136^ ^]^ On the other hand, m6A modified *lncDBET* in bladder cancer (BCa) and lncRNA *CCAT1* in LUAD can both interact with FABP5 directly to activate the PPAR signaling pathway or PPAR‐RXR transcriptional complex in order to promote lipogenesis, proliferation and migration of cancers.^[^ ^137^, ^138^ ^]^ Additionally, *circ_ZFR* in breast cancer (BC) and circPUM1 in clear cell renal cell carcinoma (ccRCC) can both absorb miRNAs (miR‐223‐3p and miR‐340‐5p, respectively) to regulate FABP7‐mediated proliferation and progression of these cancer cells.^[^ ^139^, ^140^ ^]^

FAO or β‐oxidation in mitochondria and peroxisome is the major pathway for degradation of fatty acids and production of ATP and NADPH, with CPT1 being one of the most important regulatory targets. The dysregulated lipid‐metabolic related lncRNAs and circRNAs in cancer can support tumor progression, stem cell property and drug resistance by modulating this carnitine palmitoyltransferase via various regulatory strategies (Figure 5). For instance, in MSC (mesenchymal stem cell)‐cultured breast cancer cells, overexpressed *lncRNA AGAP2‐AS1* not only binds to the CPT1 mRNA to increase its stability and expression by interacting with HuR (an RNA binding protein), but also serves as a sponge to release the miR‐15a‐5p‐mediated repression of CPT1 expression.^[^ ^141^ ^]^ Similarly, *lncRNA HCP5* is also induced in MSC‐cocultured gastric cancer cells, where it can upregulate the expression of PPARGC1A to increase the formation of PGC1α/CEBPB complex by sequestering miR‐3619‐5p. The PGC1α/CEBPB complex can further promote the expression of *CPT1* transcriptionally.^[^ ^142^ ^]^ Additionally, lncRNA *MACC1‐AS1* has been identified to be elevated in MSC‐cultured gastric cancer cells, which promotes the expression of *CPT1* directly through the *MACC‐AS1*/ miR‐145‐5p/*CPT1* axis possibly.^[^ ^143^ ^]^ In a similar way, *circ_00 24 107*, also enriched in MSC‐cultured gastric cancer cells, has been identified to mediate lymphatic metastasis of gastric cancer, in which *circ_00 24 107* can promote FAO reprogramming by modulating the *circ_00 24 107*/ miR‑5572 and miR‑6855‑5p/*CPT1A* axis.^[^ ^144^ ^]^ On the other hand, as a tumor suppressor, P53 has been recently shown to be modulated by lncRNA *SOCS2‐AS1* to enhance FAO and proliferation of papillary thyroid carcinoma. *SOCS2‐AS1* binds to P53 directly and facilitates its degradation, further stimulating FAO‐mediated cell proliferation.^[^ ^145^ ^]^

### 4.3 The lncRNAs‐CircRNAs in Regulating Lipid Esterification and Lipid Droplet Metabolism of Cancer

The metabolism of long‐chain fatty acids is dependent on their activation by esterification, which forms fatty acyl‐CoA esters from free long‐chain fatty acids catalyzed by long‐chain acyl‐coenzyme A synthetases (ACSLs) family. The ACSLs isoforms mainly involves both anabolic (lipogenesis) and catabolic pathways (fatty acid oxidation and lipolysis), among which ACSL1 and ACSL4 are the most extensively studied in the lipid metabolic reprogramming of cancer.^[^ ^146^, ^147^ ^]^ The lipid‐metabolic related lncRNAs and circRNAs in cancers have also been discovered to play roles in the regulation of ACSL1 and ACSL4 expressions (Figure 5 and Table 3). For instance, lncRNA *HULC* was identified to modulate the lipid metabolic programming of HCC. The mechanism in detail is that HULC released the miR‐9‐mediated repression of PPARA by modulating the methylation of miR‐9 promoter. The increased expression of PPARA further led to the transcriptional activation of ACSL1, resulting in triglyceride and cholesterol formation in hepatocellular carcinoma (HCC).^[^ ^148^ ^]^ Moreover, highly‐expressed lncRNA *PRADX* activated the phosphorylation of STAT3 to promote the expression of ACSL1 by suppressing the expression of BLCAP (a tumor suppressor gene) in mesenchymal glioblastoma (GBM).^[^ ^149^ ^]^ The upregulated ACSL1 further played roles in basal respiration, proton leak, and ATP production to promote energy metabolism and tumorigenesis of mesenchymal GBM cells. Additionally, the lncRNA *SNHG7*/miR‐449a/ACSL1 axis in thyroid cancer and the *circPDHX*/miR‐497‐5p/ACSL1 axis in prostate cancer have been identified to promote cancer cells’ proliferation and migration.^[^ ^150^, ^151^ ^]^

The roles of ACSL4 regulated by lipid‐metabolic related lncRNAs and circRNAs of cancer mainly involve into two biological progresses. First, ACSL4 has the basic role of long‐chain fatty acids esterification to promote tumor's proliferation, progression and drug resistance. For instance, lncRNA *NEAT1* have been identified to promote proliferation, invasion and docetaxel resistance in prostate cancer (PCa) by releasing miR‐34a‐5p and miR‐204‐5p‐mediated repression of *ACSL4* expression.^[^ ^152^ ^]^ Second, ACSL4 acts as a positive regulator in ferroptosis, a novel programmed cell death characterized by the accumulation of iron and lipid peroxidation. For example, *circSCN8A* in NSCLC and circLMO1 in cervical cancer were downregulated to inhibit the ACSL4‐mediated ferroptosis by sponging miR‐4192 and miR‐1290, respectively, facilitating the proliferation and metastasis of cancer cells.^[^ ^153^, ^154^ ^]^ Additionally, it has been discovered that the upregulation of *lncRNA CBSLR* in gastric cancer plays a protective role in cancer cells against ferroptosis, resulting in drug resistance. The mechanism involves *CBSLR* binding to YTHDF2, thus forming a *CBSLR*/YTHDF2/CBS signaling pathway that weakens the stability of CBS mRNA. This, in turn, increases the polyubiquitination and degradation of ACSL4 protein.^[^ ^155^ ^]^

Similar to fatty acids’ esterification, cholesterol can be esterified with fatty acids by ACAT1 (SOAT1) and ACAT2 to control the equilibrium between free cholesterol and cytoplasmic cholesteryl esters. These cholesterol acyltransferases have also been regulated by lipid‐metabolic related lncRNAs and circRNAs in cancer to boost their proliferation and metastasis (Figure 5). For instance, *circLDLR* has been found to be overexpressed in CRC, and can upregulate the expression of SOAT1 by sponging miR‐30a‐3p, which modulated the cholesterol levels and facilitated the malignant progress of CRC.^[^ ^156^ ^]^ Moreover, *circRPL23A* was downregulated in clear cell renal cell carcinoma (ccRCC) to promote cell proliferation, migration, and invasion by enhancing miR‐1233‐mediated repression of ACAT2 expression. But it is unknown whether these biological functions of cancer cells have relevance with the reprogramming of lipid metabolism in cancer.^[^ ^157^ ^]^ Additionally, esterification of fatty acid and cholesterol usually contribute the formation of lipid droplets (LDs) for energy storage, ROS homeostasis and entrapment of anticancer drugs in cancer cells.^[^ ^71^ ^]^ The lipid‐metabolic related lncRNAs and circRNAs in cancer have been identified to modulate the metabolism of LDs in cancer. For example, *linc01410* was overexpressed in cervical cancer to release miR‐532‐5p‐mediated repression of *PLIN2, ACC1* and *FASN* expression, which promoted LDs formation and metastasis of cervical cancer cells.^[^ ^158^ ^]^ On the other hand, lncRNA *SPRY4‐IT1* in melanoma cells can repress the accumulation of lipin2 protein and decrease the DGAT2‐mediated formation of TAG and LDs, furtherly eliminating apoptosis caused by cellular lipotoxicity.^[^ ^159^ ^]^

LDs in cancer cells are dynamic to keep the equilibrium between lipids biogenesis and lipolysis. When energy or membrane synthesis is needed, LDs in cancer cells can be rapidly lipolyzed to release free fatty acids and cholesterols, which will be further utilized as energetic sources, signaling molecules and membrane building blocks.^[^ ^65^, ^70^ ^]^ The lipid‐metabolic related lncRNAs and circRNAs have been found to play roles in the lipolysis of LDs to support the proliferation and metastasis of cancer cells (Figure 5). For example, lncRNA *NEAT1* has been identified to be overexpressed in HCC, in which *NEAT1* can promote the lipolysis of HCC via ATGL/DAG+FFA/PPARα signaling as a sponge of miR‐124‐3p.^[^ ^160^ ^]^ Moreover, when overexpressed in ovarian cancer, *NEAT1* can modulate ATGL to promote lipolysis and progression of tumor via the let7g/ MEST (mesoderm specific transcript) axis, inhibition of which can elevate the express of ATGL.^[^ ^152^ ^]^ Additionally, *circ_cMras* has been found to be down‐regulated in lung adenocarcinoma (LAC), in which *circ‐cMras* can modulate the expression of ATGL to promote tumor proliferation and aggression through the NF‐κB signaling pathway.^[^ ^161^ ^]^ On the other hand, the lipid‐metabolic related lncRNAs and circRNAs can indirectly modulate the lipolysis of LDs in cancer. For instance, *circRIC8B* has been shown to be elevated in chronic lymphocytic leukemia (CLL). Mechanically, *circRIC8B* increased the expression of lipoprotein lipase (LPL) by directly binding miR‐199b‐5p, which further promoted cellular lipoproteins uptake and the hydrolysis of triglycerides (TGs) to facilitate the progression of CLL.^[^ ^162^ ^]^ Moreover, Acyl‐CoA thioesterase 7 (ACOT7) plays a role in the lipolysis by converting Arachidonyl‐CoA to arachidonic acid and CoA. *LncRNA NMRAL2P* has been demonstrated to suppress the expression of *ACOT7* by methylating its gene's promoter via binding DNMT3b.^[^ ^163^ ^]^ Furthermore, lipid browning in cancer cells is a process of converting lipid droplets into tiny pieces, which leads to inhibiting tumor progression. Recently, *lncRNA COL18A1‐AS1* has been shown to act as a sponge of miR‐1286 to modulate the expression of transcriptional factor Krüppel‐like factor 12 (KLF12), which regulates uncoupling protein 1 (UCP1)‐mediated lipid browning in cell renal cell carcinoma.^[^ ^164^ ^]^

### 4.4 The lncRNAs‐circRNAs in Regulating Lipid Metabolic Transcriptional Factors and Oncogenic Signaling Pathways

As the major transcriptional factors of cellular lipid metabolism, SREBPs can regulate the expression of most lipogenic enzymes at the transcriptional level. The lipid‐metabolic related lncRNAs and circRNAs in cancer can modulate the expression and stability of SREBPs through various regulatory mechanisms, such as sponging miRNAs, binding protein and DNA, and affecting signaling pathways, even via modulating their regulators (Figure 6 and Table 4). For instance, *linc02570* in nasopharyngeal carcinoma and lncRNA PCA3 in prostate cancer have been demonstrated to be overexpressed and can increase the expression of SREBP1 as a sponge of miR‐4649‐3p and miR‐132‐3p, respectively.^[^ ^165^, ^166^ ^]^ Similarly, *lncRNA SNHG16* can release the miR‐195‐mediated repression of SREBP2 expression as a sponge to promote lipogenesis in pancreatic cancer cells.^[^ ^167^ ^]^ Moreover, lncRNA *HOXB‐AS3* have been shown to co‐regulate the expression of *SREBP1* with RNA‐binding protein‐PTBP1 via direct binding, which further promotes lipid metabolism and proliferation of endometrial cancer (EC).^[^ ^168^ ^]^ Another RNA‐binding protein HuR has also been found to co‐regulate the mRNA stability of *SREBP1* with *circMyc* and degradation of *Myc* with *circREOS*. *CircMyc* (hsa_circ_00 85 533) has been found to be remarkably upregulated in triple‑negative breast cancer. It maintains the mRNA stability of SREBP1 via directly binding to HuR protein.^[^ ^169^ ^]^ On the other hand, *circREOS* has been shown to directly interact with HuR protein to restrain its binding and activation of *Myc* mRNA, resulting in degradation of *My*c mRNA and restraining FASN‐mediated lipid accumulation in osteosarcoma.^[^ ^170^ ^]^ LncRNA *ZFAS1* binds to PABP2 (polyadenylate‐binding protein 2) to maintain mRNA stability of SREBP1 in CRC, which further upregulates the expression of SREBP1 and its target genes (SCD1 and FASN) to promote lipid accumulation in CRC.^[^ ^171^ ^]^ *LINC01138* interacts with PRMT5 (arginine methyltransferase 5) to methylate arginine of SREBP1 protein, sustaining the protein stability of SREBP1 and promoting lipogenesis and cell proliferation in ccRCC.^[^ ^172^ ^]^ LncRNA *CCAT1* has been identified to upregulate the expression of SREBP2 in osteosarcoma by binding to PKM2, which facilitates the phosphorylation and stability of SREBP2 protein.^[^ ^173^ ^]^ Additionally, derived from AMPK's α1 subunit, *circPRKAA1* facilitates a tetrameric complex between mature SREBP1 (mSREBP1) and the Ku80/Ku70 heterodimer by binding to Ku proteins to increase the stability of mSREBP1 in several cancer cells. Meanwhile, *circPRKAA1* can directly interact with the promoters of SREBP1 target genes to recruit mSREBP‐1, thereby promoting lipogenesis in cancer cells.^[^ ^174^ ^]^ Recently, a novel hypoxia‐induced circRNA *circINSIG1* has been found to promote the proliferation and metastasis of colorectal cancer (CRC) via enhancing cholesterol biosynthesis, in which a 121 amino acid protein encoded by *circINSIG1* (circINSIG1‐121) can promote the degradation of INSIG1 protein via the ubiquitin‐proteasome pathway and further enhancing active nuclear SREBP2 (nSREBP2)‐mediated cholesterol biosynthesis.^[^ ^175^ ^]^ On the contrary, another novel CCT3‐*LINC00326* regulatory network has been identified in hepatocellular carcinoma (HCC), in which *LINC00326* can impede CCT3's confinement of CREM/CREB1 and ATF2 to improve transcription of lipid metabolism genes. *LINC00326* itself, by binding to CCT3, facilitates EGR1, GLIPR1 and CYR6‐mediated lipogenesis decrease and lipolysis increase to inhibit tumor growth.^[^ ^176^ ^]^

In the oncogenic signaling pathways involving lipid metabolism, the PI3K‐AKT‐mTOR pathway is well studied and plays important roles in the lipogenesis of cancer. Recently, studies have demonstrated that lipid‐metabolic related lncRNAs and circRNAs are involved in regulating lipid metabolism in cancer via the signaling pathways (Figure 6). For instance, the EGFR/PI3K/AKT/NF‐κb/SREBP1 signaling pathway is activated by lncRNA *DNAJC3‐AS1* to increase the expression of lipogenesis genes in CRC, such as *ACC1* and *FASN*.^[^ ^177^ ^]^ LncRNA *HAGLROS* has also been found to improve the lipid metabolism in intrahepatic cholangiocarcinoma via the mTOR/SREBP1 axis. However, its relevant mechanism is need to be explored in future studies.^[^ ^178^ ^]^ Moreover, *linc01468* is overexpressed in non‐alcoholic fatty liver disease (NAFLD) associated HCC and adjacent samples to facilitate the proliferation of HCC through modulating lipogenesis, in which *linc01468* binds SHIP2 protein to induce CUL4A by promoting the ubiquitinated degradation of SHIP2, thereby activating the Akt/mTOR signaling pathway mediated‐increase of intracellular triglyceride (TG), and total cholesterol (TC).^[^ ^179^ ^]^ Similarly, *lncARSR* was found to be elevated in NAFLD associated HCC to promote proliferation, migration and lipid accumulation in HCC through activating the IRS2/AKT pathway, in which *lncARSR* binds with YAP1 protein to inhibit its phosphorylation nuclear translocation.^[^ ^180^ ^]^ Additionally, lncRNA *NEAT1* has been identified to bind with TRIM25 to reduce the degradation of RPRD1B protein, which transcriptionally upregulates c‑Jun/c‑Fos and activates the c‑Jun/c‑Fos/SREBP1 axis to enhance fatty acid uptake and synthesis in gastric cancer.^[^ ^181^ ^]^ On the contrary, *lncHR1* has an opposite effect on the regulation of SREBP‐1c‐mediated fatty acid synthesis in HCC, in which *lncHR1* suppresses the phosphorylation of the DK1/AKT/FoxO1 axis to weaken the combinatorial capacity of LXRα/RXR binding to LXREs in the promoter of SREBF1 gene, thereby decreasing the expression of SREBP‐1c and lipogenesis.^[^ ^182^ ^]^

On the other hand, the lipid‐metabolic related lncRNAs and circRNAs can modulate FAO pathway through various signaling pathways in cancer (Figure 6). For example, as the antisense RNA of *SLC16A1/MCT1* (protein‐coding gene), lncRNA *SLC16A1‐AS1* can interact with transcription factor‐E2F1 to form an RNA‐protein complex in E2F1‐driven aggressive bladder cancer, which further enhances the expression of the key mediator of fatty acid β‐oxidation‐PPARA through binding the SLC16A1‐AS1:E2F1‐responsive element in PPARA's promoter.^[^ ^183^ ^]^ As an oncogene, *Linc00924* has been found to promote the invasion and metastasis of peritoneal metastasis‐associated gastric cancer. The mechanism involves *Linc00924* binding to hnRNPC to regulate the alternative splicing of Mnk2 pre‐mRNA. Consequently, Mnk2a splicing is reduced, leading to the activation of the p38 MAPK/PPARα signaling pathway, which in turn facilitates fatty acids oxidation (FAO) and uptake.^[^ ^184^ ^]^ Moreover, under metabolic stress in CRC cells, *circACC1*, derived from human ACC1 mRNA, can bind the regulatory β and γ subunits of AMPK to stabilize and promote the holoenzyme activity of AMPK, which can repress fatty acid synthesis (FAS) and stimulates FAO by the phosphorylation of ACC1 and ACC2, respectively.^[^ ^185^ ^]^ Additionally, a novel 113‐amino acid protein (p113), derived from circRNA *CUX1*, have been recently induced under serum deprivation in neuroblastoma. P113 can further form a transcriptional regulatory complex with ZRF1 and BRD4 to promote the conversion of fatty aldehydes into fatty acids, mitochondrial complex I and fatty acid β‐oxidation.^[^ ^186^ ^]^

## 5 Relevance of Lipid Metabolic Related lncRNAs‐circRNAs to Other Phenotypes of Cancer

With development of RNA‐seq technologies and bioinformatics analysis, more and more lipid‐metabolic related lncRNAs and circRNAs have been uncovered to play roles in several phenotypes of cancer by regulating lipid metabolism. These roles include facilitating progression and metastasis, inducing angiogenesis and lymphangiogenesis, sustaining stemness and chemotherapy resistance, among others.

### 5.1 The Lipid Metabolic Related lncRNAs‐circRNAs in Promoting Proliferation and Metastasis of Cancer

As one of the hallmarks of cancer, lipid‐metabolic reprogramming has been well explored in an increasing number of studies, which revealed that the lipid anabolism and catabolism (such as *de novo* lipids synthesis and fatty acid oxidation) are reprogrammed to provide building blocks of membrane biosynthesis, energy source, and signaling molecules for rapid growth and migration in cancer.^[^ ^36^, ^187^ ^]^ Therefore, most lipid‐metabolic related lncRNAs and circRNAs in cancer have been identified to play roles in promoting cancer rapid proliferation, invasion, and metastasis. For instance, lncRNA *CTD‐2245E15.3* in NSCLC not only has been associated with multiple protein‐coding genes involved in tumor growth, but also has been demonstrated to facilitate tumor progression by enhancing ACC1‐mediated lipogenesis.^[^ ^106^ ^]^ Moreover, *circWHSC1*, overexpressed in breast cancer, promoted cell growth, invasion, and metastasis via miR‐195‐5p/FASN/AMPK/mTOR axis to upregulate N‐cadherin, Vimentin, and Bcl2 and inhibit E‐cadherin, BAX and c‐Caspase3 expression.^[^ ^111^ ^]^ Furthermore, lncRNA *ZFAS1* was upregulated in CRC tissues and cell lines, in which *ZFAS1* promoted proliferation and metastasis of CRC in vitro and vivo through modulating SREBP1/SCD1&FASN‐mediated lipogenesis.^[^ ^171^ ^]^ Additionally, m6A‐induced *lncDBET* has been identified to promote the proliferation and migration of bladder cancer by modulating the FABP5/PPAR signaling pathway‐mediated lipid metabolism.^[^ ^137^ ^]^ Another lncRNA *TINCR* was found to be significantly upregulated in nasopharyngeal carcinoma, where *TINCR* promoted de novo lipogenesis, proliferation, and metastasis of nasopharyngeal carcinoma via ACLY‐PADI1‐MAPK‐MMP2/9 pathway.^[^ ^103^ ^]^

### 5.2 The Lipid Metabolic Related lncRNAs‐circRNAs in Angiogenesis and Lymphangiogenesis of Cancer

Angiogenesis refers to the biological process by which new blood vessels are developed from pre‐existing ones, primarily through the involvement of endothelial cells. The principal drivers of this process are tyrosine kinases such as vascular endothelial growth factor‐A (VEGF‐A) and the VEGF receptor (VEGFR). VEGF expression in cancer is usually mediated by multiple regulators, such as loss of p53 and VHL function or activation of Ras oncogene.^[^ ^188^, ^189^ ^]^ Recent evidence demonstrates that lipid‐metabolic‐related lncRNAs and circRNAs in cancer can also regulate tube formation of HUVECs in cancer tissues via lipid metabolism reprogramming. For instance, lncRNA *CCAT1* can activate the expression of VEGFA to facilitate the angiogenesis of lung adenocarcinoma by binding FABP5 to translocate fatty acid into nuclear and inducing PPAR/RXR transcriptional complex.^[^ ^138^ ^]^ Additionally, *circ_0 0 08078* can inhibit the tube formation ability of esophagus cancer via interfering the ELOVL4‐mediated lipid metabolism.^[^ ^118^ ^]^

Lymphangiogenesis, like angiogenesis, involves the formation of new vessels, but in this case, it is new lymphatic vessels forming from pre‐existing ones. This process is considered an important step in lymph node metastasis in cancer. Vascular endothelial growth factor‐C and ‐D (VEGF‐C and VEGF‐D) have been identified as the primary factors responsible for lymphangiogenesis, acting through the VEGFR‐3 receptor and contributing to lymph node metastasis in cancer.^[^ ^190^, ^191^ ^]^ Similarly, lipid metabolic related lncRNA and circRNAs in cancer have been demonstrated to involve into this process through modulating lipid metabolism. For example, *LNMICC* has been identified to promote lymph node metastasis in cervical cancer by modulating FABP5‐mediated fatty acid metabolism and elevating the expression of VEGF‐C.^[^ ^135^ ^]^ Additionally, lncRNA *NEAT1* was uncovered to promote lymph node metastasis in gastric cancer through facilitating RPRD1B‐mediated fatty acid metabolism by the c‐Jun/c‐Fos/SREBP1 axis.^[^ ^181^ ^]^

### 5.3 The Lipid Metabolic Related lncRNAs‐circRNAs in Stemness Maintenance and Chemotherapy Resistance of Cancer

Acquisition of stemness property in cancer cells is an important mechanism in chemotherapy resistance, elicited by mesenchymal stem cells (MSCs) in tumor environment.^[^ ^192^ ^]^ Fatty acid oxidation has been shown to support stemness property and chemotherapy resistance in multiple cancer cells, provoked by MSCs.^[^ ^193^ ^]^ The lipid‐metabolic related lncRNAs and circRNAs have recently been viewed as important participants in this process. For instance, lncRNA *HCP5*, induced by MSCs in gastric cancer (GC), has been found to play a role in promoting stemness and chemoresistance of GC cells. This is achieved through the regulation of fatty acid oxidation (FAO) via the miR‐3619‐5p/PGC1α/CEBPB/CPT1 axis.^[^ ^142^ ^]^ Moreover, lncRNA *MACC1‐AS1* was upregulated in GC cells co‐cultured with MSCs through the TGF‐β/ TGF‐β receptor/SMAD2/3 axis. *MACC1‐AS1* then promoted the FAO‐dependent stemness property and chemotherapy resistance of GC.^[^ ^143^ ^]^ Furthermore, lncRNA *AGAP2‐AS1* was overexpressed to promote stemness and trastuzumab resistance in breast cancer co‐cultured with MSCs via CPT1‐mediated FAO.^[^ ^141^ ^]^ Cholesterol biosynthesis has been shown to be of importance in maintaining the stemness property and chemotherapy resistance of cancer stem cells (CSCs). Apart from being an important component of cell membranes and lipid rafts, cholesterol can act as a mitogen for stemness and chemoresistance in CSCs through activating the PI3K/AKT or MAPK signaling pathways, as well as the Notch and Wnt canonical signaling pathways. Additionally, the mevalonate pathway (MVA) in cholesterol *de novo* biosynthesis is the only source of farnesyl‐diphosphate and geranylgeranyl‐diphosphate (GGPP), which is important for prenylation of RasGTPase superfamily, including Ras and Rho, and the Rho‐dependent YAP/TAZ nuclear localization to facilitate stemness genes expression in CSCs.^[^ ^194^, ^195^ ^]^ For instance, *Lnc30* has been identified to enhance the biosynthesis of cholesterol that is mediated by SQLE. This process then leads to the activation of the PI3K/Akt signaling and ultimately regulates the stemness of breast cancer stem cells (BCSC).^[^ ^128^ ^]^ Phospholipid metabolism‐related *lncROPM* has been recently identified to promote PLA2G16‐mediated phospholipid lipolysis and the release of free fatty acid, which activates the PI3K/AKT, Wnt/β‐catenin, and Hippo/YAP signaling pathways, thereby involving in the maintenance of breast CSCs stemness.^[^ ^196^ ^]^

Apart from enhancing FAO, inhibiting ferroptosis emerges as a novel strategy to resist chemotherapy in multiple cancers, where lipid‐metabolic related lncRNAs and circRNAs have been identified to play important roles. For instance, hypoxia‐induced lncRNA *CBSLR* has been uncovered to protect gastric cancer from ferroptosis‐dependent chemotherapy through increasing ACSL4 protein degradation via forming CBSLR/YTHDF2/CBS complex under a hypoxic microenvironment.^[^ ^155^ ^]^ Moreover, *circLMO1* has been revealed to be downregulated to facilitate cervical cancer cell proliferation and metastasis through reducing ACSL4‐dependent ferroptosis.^[^ ^153^ ^]^ Furthermore, exosomal *circRNA_101 093 (cir93)* can interact with FABP3 to stimulate arachidonic acid (AA) transportation and interaction with taurine, which helps to desensitize ferroptosis‐dependent treatment in lung adenocarcinoma.^[^ ^133^ ^]^

## 6 Conclusion and Perspective

Lipid metabolism is reprogrammed in various types of cancers under different metabolic conditions and during distinct stages of tumorigenesis. In this review, lipid‐metabolic related lncRNAs and circRNAs have been uncovered to involve into almost every aspect of lipid metabolic reprogramming in cancer, from lipogenesis to lipolysis, which further to support the formation of other hallmarks in cancer through reprogrammed lipid metabolism, including progression and metastasis, angiogenesis and lymphangiogenesis, stemness and chemotherapy resistance. These lipid‐metabolic related lncRNAs and circRNAs not only greatly strengthen the interplay between lncRNAs/circRNAs and lipid metabolic reprogramming in cancer, but also enlarge the association between lipid metabolic reprogramming with other hallmarks of cancer (Tables 1, 2, 3, 4).

On the one hand, a small part of lipid‐metabolic related lncRNAs and circRNAs have been found to act as the mediators involving into the regulation of lipid metabolic participants to promote cancer progression, such as the *circWHSC1*/miR‐195‐5p/FASN axis in breast cancer,^[^ ^111^ ^]^ the *CASC19*/miR‐301b‐3p/LDLR axis in Non‐small cell lung cancer (NSCLC),^[^ ^131^ ^]^ and the *NEAT1*/miR‐34a‐5p&miR‐204‐5p/ACSL4 axis in prostate cancer.^[^ ^152^ ^]^ On the other hand, most of lncRNAs and circRNAs summarized in this review are responsible for the lipid metabolic reprogramming of cancer to facilitate other hallmarks of cancer (**Table** **5**). For instance, *circCAPRIN1* promotes proliferation, metastasis and EMT of colorectal cancer (CRC) through regulating ACC1‐mediated lipogenesis.^[^ ^105^ ^]^ *Lnc030* promotes the stemness of breast cancer stem cells (BCSC) by cooperating with poly(rC) binding protein 2 (PCBP2) to facilitate the SQLE‐mediated cholesterol synthesis.^[^ ^128^ ^]^ *CircINSIG1* has been found to promote the proliferation and metastasis of CRC via enhancing SREBP2‐mediated cholesterol biosynthesis.^[^ ^175^ ^]^ However, there are still a lot of unknown lncRNAs and circRNAs that are needed to be identified in lipid metabolic reprogramming of cancer. Therefore, more and more new lipid‐metabolic related lncRNAs and circRNAs mediating or responsible for lipid metabolic reprogramming in cancer will be discovered in the future, with the progression of RNA sequencing and metabonomics.

**Table 5:** The lipid‐related lncRNAs‐circRNAs in cancers' lipid‐metabolic reprogramming.

Cancer types	De novo synthesis of fatty acid	Fatty acid desaturation	De novo synthesis of cholesterol	Lipid transport	Fatty acid oxidation	Esterification of lipids	Metabolism of lipid drops (LDs)	Transcriptional factors SREBPs	Oncogenic signaling pathways
Neuroblastoma (NB)	‐	‐	‐	FAM201A/ FABP5/ FABP5	‐	‐	‐	‐	EcircCUX1/ ZRF1/ ALDH3A1
Glioma	‐	‐	‐	Circ_ABCB10/ miR‐620/ FABP5	‐	PRADX/ EZH2/ ACSL1	‐	‐	‐
Nasopharyngeal carcinoma (NPC)	TINCR/ ACLY/ acetyl‐CoA; HAGLR/ N.A/ FASN	LINC01569/ miR‐193a‐5p/ FADS1	‐	‐	‐	‐	‐	Linc02570/ miR‐4649‐3p and miR‐132‐3p/ SREBP1	‐
Non‐small cell lung cancer (NSCLC)	CTD‐2245E15.3/ ACC1/ ACC1; HOTAIR/ N.A/ / FASN; CircFARSA/ miR‐330‐5p&miR‐326/ FASN	‐	‐	CASC19/ miR‐301b‐3p/ LDLR	‐	CircSCN8A/ miR‐1290/ ACSL4	‐	‐	‐
Lung adenocarcinoma (LUAD)	‐	‐	‐	Circ_101 093/ FABP3/ FABP3; CCAT1/ FABP5/ PPAR/ RXR	‐	‐	Circ_cMras/ N.A/ ABHD5/ ATGL	‐	‐
Breast cancer (BC)	CircWHSC1/ miR‐195‐5p/ FASN CircZFAND6/ miR‐647/ FASN	‐	Lnc030/ PCBP2/ SQLE	Circ_ZFR/ miR‐223‐3p/ FABP7	AGAP2‐AS1/ HuR&miR‐15a‐5p/ CPT1	‐	‐	CircMyc/ HuR&Myc/ SREBP1	‐
Thymic epithelial tumors (TETs)	‐	Linc00174/ miR‐145‐5p/ SCD5	‐	‐	‐	‐	‐	‐	‐
Thyroid cancer (TC)	‐	‐	‐	‐	SOCS2‐AS1/ P53/ P53 turnover	SNHG7/ miR‐449a/ ACSL1	‐	‐	‐
Hepatocellular carcinoma (HCC)	FASRL/ ACACA/ ACACA	‐	AT102202/ N.A/ N.A	BM450697/ LDLR promoter/ LDLR	‐	HULC/ miR‐9/ ACSL1	NEAT1/ miR‐124‐3p/ ATGL	CircPRKAA1/ Ku80/ Ku70/ mSREBP1	lncHR1/ N.A/ DK1/ AKT/ FoxO1; Linc01468/ SHIP2/ CUL4A; lncARSR/ YAP1/ IRS2/ AKT pathway
Intrahepatic cholangiocarcinoma (ICC)	CircMBOAT2/ PTBP1/ FASN	‐	‐	‐	‐	‐	‐	‐	HAGLROS/ N.A/ mTOR pathway
Esophagus cancer (EC)	Circ_0 0 08078/ miR‐191‐5p/ ELOVL4	KDM4A‐AS1/ N.A/ SCD, FASN	‐	‐	‐	‐	‐	‐	‐
Gastric cancer (GC)	FLJ22763/ N.A/ N.A; Circ_0000182/ miR‐579‐3p/ SQLE	‐	‐	‐	LncHCP5/ miR‐3619‐5p/ CPT1; MACC1‐AS1/ miR‐145‐5p/ FAO genes; Circ_00 24 107/ miR‐5572&miR‐6855‐5p/ CPT1A	CBSLR/ YTHDF2/ ACSL4	NMRAL2P/ N.A/ ACOT7	‐	NEAT1/ TRIM25/ c‐Jun/ c‐Fos/ SREBP1; Linc00924/ hnRNPC/ p38 MAPK/ PPARα signaling pathway
Pancreatic carcinoma (PC)	Circ_00 18 909/ miR‐545‐3p/ FASN	‐	ZFAS1/ U2AF2/ HMGCR	‐	‐	‐	‐	SNHG16/ miR‐195/ SREBP2	‐
Colorectal cancer (CRC)	TSPEAR‐AS2/ N.A/ N.A; CircCAPRIN1/ STAT2/ ACC1	UPAT/ UHRF1/ SPRY4&SCD1; SNHG16/ 26 unique miRNAs/ SCD	‐	TINCR/ miR‐107/ CD36	‐	CircLDLR/ miR‐30a‐3p/ SOAT1	‐	ZFAS1/ PABP2/ SREBP1; CircINSIG1/ INSIG1/ SREBP2	DNAJC3‐AS1/ EGFR/ PI3K/ AKT/ NF‐Kb/ SREBP1; CircACC1/ AMPK β, γ/ AMPK
Clear cell renal cell carcinoma (ccRCC)	‐	‐	‐	CircPUM1/ miR‐340‐5p/ FABP7	‐	CircRPL23A/ miR‐1233/ ACAT2	COL18A1‐AS1/ miR‐1286/ KLF12	Linc01138/ PRMT5/ SREBP1	
Bladder cancer (BCa)	‐	‐	‐	LncDBET/ FABP5/ PPAR		‐	‐	‐	SLC16A1‐AS1/ E2F1/ PPARA
Endometrial cancer (EC)	SNHG25/ miR‐497‐5p/ FASN	‐	‐	‐	‐	‐	‐	HOXB‐AS3/ PTBP1/ SREBP1	‐
Cervical cancer (CC)	‐	‐	‐	LNMICC/ NPM1/ FABP5	‐	CircLMO1/ miR‐4192/ ACSL4	Linc01410/ miR‐532‐5p/ FASN	‐	‐
Ovarian cancer (OC)	‐	‐	‐	‐	‐	‐	‐	NEAT1/ Let‐7 g/ MEST	‐
Prostate cancer (PCa)	‐	‐	‐	‐	‐	CircPDHX/ miR‐497‐5p/ ACSL1; NEAT1/ miR‐34a‐5p&miR‐204‐5p/ ACSL4	‐	PCA3/ miR‐4649‐3p and miR‐132‐3p/ SREBP1	‐
Chronic lymphocytic leukemia (CLL)	‐	‐	‐	‐	‐	‐	‐	CircRIC8B/ miR‐199b‐5p/ LPL	‐
Melanoma	‐	‐	‐	‐	‐	‐	SPRY4‐IT1/ lipin2/ DGAT2	‐	‐
Osteosarcoma (OS)	PVT1/ miR‐195/ FASN	Linc00460/ miR‐1224‐5p/ FADS1; Circ_0000073/ miR‐1184/ FADS2	‐	‐	‐	‐	‐	CCAT1/ PKM2/ SREBP2; CircREOS/ HuR/ Myc	‐

PS: Format: lncRNA or circRNA/Interact/Target; – : None; N.A.: Not Available.

As the novel noncoding RNAs, lncRNAs and circRNAs have been shown to play diverse roles in regulating gene expression, acting as signal or indicator, guide, decoy, and scaffold, even as translators for protein/peptide. The lipid‐metabolic related lncRNAs and circRNAs in this review have also been shown to regulate lipid metabolic reprogramming of cancer in similar ways. For instance, *circPRKAA1* can act as a guide to recruit mSREBP‐1 on the promoters of SREBP1's target.^[^ ^174^ ^]^ LncRNA *BM450697* can act as an indicator of inhibiting the interaction between the promoter of LDLR with RNA polymerase II or transcription factor SPREBP1a.^[^ ^132^ ^]^ LncRNA *SLC16A1‐AS1* can directly interact with transcription factor‐E2F1 as scaffold to form an RNA‐protein complex in E2F1‐driven aggressive bladder cancer.^[^ ^183^ ^]^ At the same time, circRNA *CUX1* acts as a translator to be translated into a novel p113 protein that promotes fatty acid β‐oxidation under serum deprivation in neuroblastoma.^[^ ^186^ ^]^ Nevertheless, these roles played by lipid‐metabolic related lncRNAs and circRNAs mentioned above are fresh and interesting, but relatively less reported in recent studies. In fact, most of lipid‐metabolic related lncRNAs and circRNAs have recently been discovered to regulate the lipid metabolic reprogramming in cancer by decoying miRNAs. With the development of RNA sequencing and extensive exploration in future, more and more regulative mechanisms of lipid‐metabolic related lncRNAs and circRNAs in nucleus will be uncovered, such as chromatin modification, mRNA splicing, as well as translation regulation, and more.

In addition, considering the important roles of lipid metabolism in cancer, most of lipid‐metabolic related lncRNAs and circRNAs have been shown to be relevant with pathological and clinical prognostic features of cancer, such as tumor size, tumor grade and TNM stage, PFS and OS. They have been viewed as new and potential diagnostic and prognostic biomarkers for cancer patients (presented in Tables 1, 2, 3, 4). Since the antisense oligonucleotide (ASO) or CRISPR/Cas9‐based strategies in vivo and RNAi‐based drugs have begun to be tested in some clinical trials, lncRNAs and circRNAs increasingly emerge as novel therapeutic targets for cancer patients.^[^ ^9^, ^11^, ^85^ ^]^ Therefore, it is worth being expected that these lipid‐metabolic related lncRNAs and circRNAs‐based diagnostics and therapeutics may one day be beneficial for cancer patients, with the rapid development of life science.

## Conflict of Interest

The authors declare no conflict of interest.

## Author Contributions

S.L. and B.J. contributed equally to this work and shared joint first authorship. S.L. and B.J. wrote and edited the manuscript, figures and tables. H.Z. and X.L. collected and prepared the related papers. F.J. reviewed and made significant revisions to the manuscript. J.H. and X.L. provided direction and guidance throughout the preparation of this manuscript. All authors read and approved the final manuscript.
